# A cyber-physical system to design 3D models using mixed reality technologies and deep learning for additive manufacturing

**DOI:** 10.1371/journal.pone.0289207

**Published:** 2023-07-27

**Authors:** Ammar Malik, Hugo Lhachemi, Robert Shorten

**Affiliations:** 1 Department of Electrical and Electronic Engineering, University College Dublin, Dublin, Ireland; 2 L2S, CentraleSupélec, Gif-sur-Yvette, France; 3 Dyson School of Design Engineering, Imperial College London, London, United Kingdom; Universiti Sains Malaysia, MALAYSIA

## Abstract

I-nteract is a cyber-physical system that enables real-time interaction with both virtual and real artifacts to design 3D models for additive manufacturing by leveraging mixed-reality technologies. This paper presents novel advances in the development of the interaction platform to generate 3D models using both constructive solid geometry and artificial intelligence. In specific, by taking advantage of the generative capabilities of deep neural networks, the system has been automated to generate 3D models inferred from a single 2D image captured by the user. Furthermore, a novel generative neural architecture, SliceGen, has been proposed and integrated with the system to overcome the limitation of single-type genus 3D model generation imposed by differentiable-rendering-based deep neural architectures. The system also enables the user to adjust the dimensions of the 3D models with respect to their physical workspace. The effectiveness of the system is demonstrated by generating 3D models of furniture (e.g., chairs and tables) and fitting them into the physical space in a mixed reality environment. The presented developmental advances provide a novel and immersive form of interaction to facilitate the inclusion of a consumer into the design process for personal fabrication.

## Introduction

**Fig 1:**
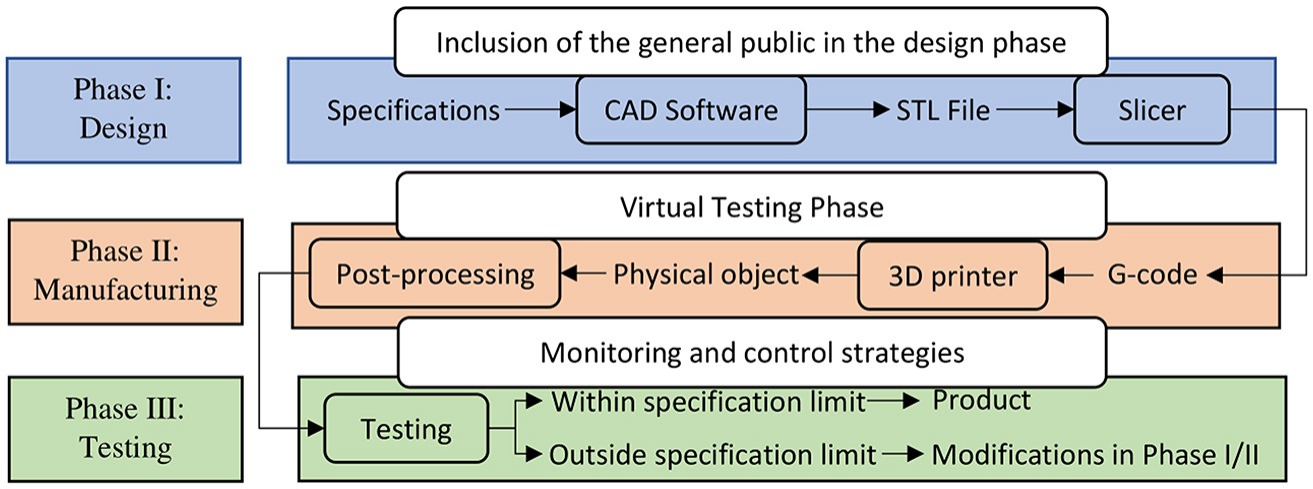
Potential improvements in the AM workflow [11].

Industry 4.0 is a digital industrial revolution in which numerous emerging technologies are converging to provide digital solutions to achieve mass customisation with increased speed, better quality, and improved productivity [1, 2]. Additive manufacturing (AM), one of the main driving forces in the realisation of this fourth industrial revolution, has emerged during the last decade as a key enabling technology poised to deeply transform manufacturing [3–5]. AM, also known as 3D printing, rapid prototyping, or generative manufacturing, refers to depositing successive thin layers of materials upon each other in precise geometric shapes based on 3D model files to manufacture three-dimensional physical objects [6]. A workflow of AM, depicted in Fig 1, consists of three phases [7]. It starts with the three-dimensional virtual model of the desired product designed via a computer-aided design (CAD) tool or obtained from 3D scanning in the design phase. Then, during the manufacturing phase, the 3D printer builds the physical object layer upon layer and post-processing is done either to remove support structures or to give the finishing touch to the 3D-printed product. Finally, the manufactured product is inspected for the desired quality and conformance during the testing phase. Therefore, in such a workflow, testing of the designed 3D model for the desired functionality is postponed to the end of the printing process. Hence, the entire loop is reiterated through a trial-error procedure until the desired results are achieved, making the design process costly and time-consuming. Moreover, most CAD design software programs not only require professional training but also restrain the design of 3D virtual models to 2D interfaces, making the design process unintuitive and cumbersome for non-technical consumers and, hence, limiting their involvement in the design phase to facilitate customisation [8, 9]. In this context, innovations in the design of CPS and technological advancements in its supporting tools (IoT, mixed reality, cloud computing, robotics, machine learning) are playing an important role in the widespread adoption of AM by the general public as well as the industry [10].

I-nteract [12] is a CPS that enables the user to interact with both the virtual as well as the physical objects (deformable and non-deformable) simultaneously in a visio-haptic mixed reality (VHMR) environment. The system streamlines the AM process by allowing the user to generate digital twins of the real objects and to test the properties of the designed virtual model in response to human and physical objects stimuli prior to printing. Hence, adding a virtual model testing phase between the design and the manufacturing phase as illustrated in Fig 1. Such innovations in the development of CPS are not only enabling the development of intuitive interfaces for human-machine interactions (human-in-the-loop) [13–15] but also provide innovative monitoring solutions to improve the build quality of the product [16, 17].

With the emergence of Industry 4.0, the horizon of product creation is shifted towards AI-enabled human-centred design innovations from merely a physical production perspective [18]. Hence, directing the product design approach towards coordinated product development to achieve customisation and end-user satisfaction enacted through human-centred cyber-physical systems (CPS) [19]. In comparison to traditional (subtractive and formative) manufacturing, AM allows the manufacturing of complex geometries without using traditional dies, molds, milling, and machining which are expensive and time-consuming for mass customization [20]. This advantage over traditional manufacturing makes AM a key enabler in producing moderate to mass quantities of products that can be customized individually for personal fabrication [6]. Although existing solutions [12–15] provide innovative interfaces to bridge the gap between the consumer, the designer, and the production using AM but only allow either modifications in the existing 3D model or 3D scanning of an existing real object. In this context, there is a need for interfaces that, along with providing an immersive experience in the three-dimensional workspace, also enable non-technical users to design 3D models from scratch with minimum effort. Constructive solid geometry (CSG) and machine learning (ML) can play a significant role to achieve this objective. CSG, also known as building block geometry, offers simple, precise, and concise methods for generating 3D models [21]. Recent developments in the generative networks [22–31], a subbranch of deep learning (DL), provide an effective solution to automate the parts of the design process that require expert knowledge for generating 3D models.

In this paper, I-nteract 2.0, an advanced development of its predecessor I-nteract [12], has been presented. The novel integration of the system with CSG and DL enables generative CAD in mixed reality (MR) for AM. A novel 3D model generative network, named SliceGen, has been proposed allowing the system to infer the genus of the object. In addition to this, the system also exploits the immersive feature of MR by enabling the user to adjust the dimensions of the virtual model with respect to the design constraints in the physical workspace. The presented developmental advances provide a novel and immersive form of interaction to facilitate the inclusion of a consumer into the design process for personal fabrication.

The remainder of this paper is structured as follows. Related works are presented in Section II. After a general description of the system in Section III, the methods to generate 3D models using I-nteract 2.0 are illustrated in Section IV. Results are reported in Section V. Finally, concluding remarks are provided in Section VI.

## Related works

The technological advancements in the areas of MR, robotics, computer vision, and ML have already enabled the development of many intuitive and realistic interfaces for humans to interact with both the physical and digital world in real time. Further, in recent years, extensive research has been devoted to improving the real-time representation of the virtual world in users’ physical environment using these innovative technologies [10]. The present section focuses on the research endeavours of such novel interfaces in the context of 3D modelling for AM.

Window-Shaping [32] is an augmented reality (AR) interface with the objective of integration of physical objects into the design process. The interface consists of a hand-held device to enable the user to perform sketch-based 3D modelling in reference to physical artifacts. Although window-shaping merges the digital and the physical worlds but provides a 2D view of a three-dimensional workspace. Modern MR solutions remedy this either by stereoscopic projections or head-mounted displays (HMDs) which also allow the user to use the hands in three-dimensional space for interaction hence enabling a more immersive experience. MirageTable [33], a freehand interactive system utilises a depth camera, a curved screen, and a stereoscopic projector to provide an MR interface for 3D modelling using gestures. Interactive situated AR systems like HoloDesk [14], Holo TableTop [15], and MixFab [13] provide intuitive interfaces to enable personal fabrication for non-technical designers. MixFab along with a depth camera for hand gestures detection and an MR display consists of a motorized turntable to enable 3D scanning of a physical object. The user then can use the scanned virtual model as a size or shape reference to design 3D models. Tangible interaction with intangible objects in an immersive augmented environment makes the experience more realistic which cannot be achieved by relying solely on visual feedback and gestures. For improving interactivity, interfaces like Surface Drawing [34], Twister [35], Digits [36], and NormalTouch and TextureTouch [37] make use of additional hardware (such as haptic gloves) for force feedback to enable physical interaction with virtual artifacts. I-nteract [12] is a VHMR system that comprises MR glasses for visual feedback, haptic glove for force feedback, and force sensors to enable dynamic interaction between human, physical, and virtual objects to streamline the design process for AM.

ML, a subset of AI, is a powerful tool that enables the system to learn automatically from data without being explicitly programmed to perform a task. Researchers are actively involved in exploring innovative ways to integrate ML within the AM process. In recent years, ML has proven to be useful in improving product quality, optimizing manufacturing processes, and reducing costs [38]. DL, a subset of ML, has emerged as an active research area to enable generative design. Generative design is an iterative design exploration process that involves the automatic generation of design options to meet certain constraints. These options are presented to the designer to fine-tune. This automated generation makes it feasible for non-technical and inexperienced users to implement their ideas. Generative design has also been integrated into many commercially available CAD packages such as Ansys (https://www.ansys.com/), Autodesk (https://www.autodesk.com/), etc. A generative design framework generates outputs that are not only aesthetic but also satisfy engineering constraints. Generative modelling is an active research area of DL that has great potential to improve generative design [39]. Generative models although not yet used to their full potential to produce engineering designs [40] but have already proven themselves to be immensely capable of inferring 3D shapes from 2D images. Variational autoencoders (VAEs) [41] and generative adversarial networks (GANs) [42] are the two significant types of generative deep convolution neural networks (CNNs) that have been extensively researched to perform generative tasks [22–31, 43, 44].

With the technological advancements in MR technologies along with the democratization of 3D printers, generative modelling using deep neural networks (DNN) has emerged as a promising tool to generate 3D models for AM [25]. 3D models have various forms of representations which lead to different DNN architectures. Volumetric (voxelized), mesh, and point cloud are the most popular and widely used 3D model representations. Each representation has its own merits when used in generative modelling. Although volumetric representation enables the 3D CNNs, a direct extension of 2D CNNs, for 3D content generation but is computationally wasteful as most information of a 3D shape lies on the surface hence making the extra third dimension redundant. Mesh and point cloud representations provide compact encoding of shape information but suffer from dimensional variability per 3D shape sample that complicates the application of learning methods to infer 3D shapes from 2D images [26]. Generative modelling using template mesh deformation [22, 23, 45] is an innovative solution to deal with this problem. As mesh representation (using triangular meshes) is predominantly used for 3D models representation both in MR and AM, therefore, the generative DNNs based on mesh representation of 3D models are more compatible to be integrated within the MR-based AM design process. The common mesh representation based 3D file formats are OBJ and STL. These file formats contain information about the vertices and the faces of the triangles to estimate the 3D shapes. In the template mesh deformation method, the DNN learns the displacement in the position of the vertices to synthesize a 3D model with respect to the input image. In this method, the number of vertices and the faces remain constant which solves the inherent problem of dimensional variability per 3D shape sample in using mesh representation. The generation of a 3D model based on a single 2D image is termed as single-view mesh reconstruction in literature. To take advantage of the generative capability of the DNNs, two generative DNNs (SoftRas [22] and SliceGen) have been integrated with I-nteract [12] for single-view mesh reconstruction in an MR environment.

## System overview

**Fig 2:**
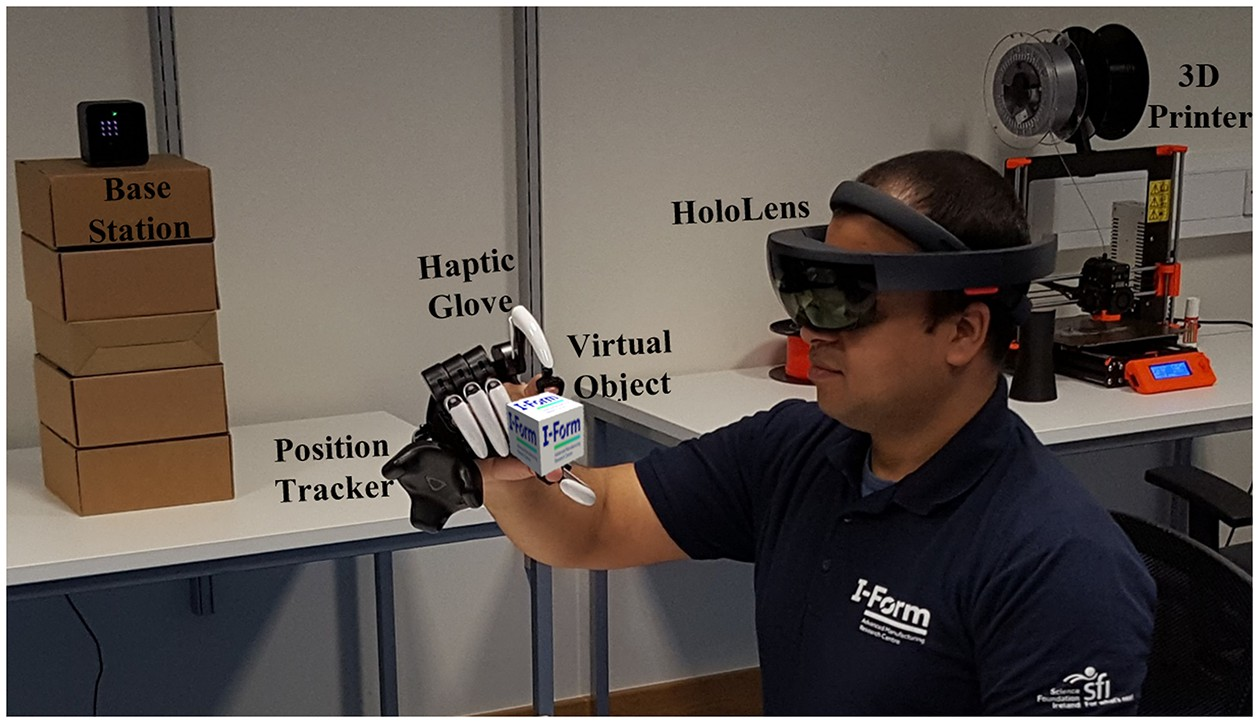
I-nteract [12].

I-nteract utilises MR and haptic feedback to provide the user with an integrated visio-haptic experience to design 3D models for AM [12]. I-nteract allows the designers to inspect and perfect virtual objects in real-time based on the interaction with other objects or humans prior to printing, and in this way streamlines the AM process. The system is built using MR smartglasses (HoloLens—https://learn.microsoft.com/en-us/hololens/hololens1-hardware) for visual feedback, haptic gloves (Dexmo—https://www.dextarobotics.com) for force feedback, and VIVE (https://www.vive.com/ca/vive-tracker/) hardware for global position tracking of the hand (glove). Hence, I-nteract provides an intuitive novel MR interface to 3D scan a physical object and to measure its physical properties (such as elasticity) to generate a digital twin. The interaction of a user with a virtual object using I-nteract is illustrated in Fig 2. The individual in Fig 2 has given written informed consent (as outlined in the PLOS consent form) to be depicted in the illustration.

### Implementation details

In this paper, further developmental advances in the VHMR system, (I-nteract) reported in [12], have been presented to enable CAD in MR for AM by using CSG and AI. To the best of our knowledge, I-nteract 2.0 is the first VHMR system that enables generative AI-based CAD in MR for AM. Integration with CSG allows the user to design 3D models from scratch using primitive 3D objects (such as cuboids, cylinders, spheres, etc.) and his/her creative skills in an MR environment. The CSG for creating meshes in MR using boolean operations has been adapted from [46]. The AI network integration enables the user to generate 3D models automatically by taking pictures of the objects using HoloLens. Although HoloLens is a self-contained computing machine but additional hardware, called Graphical Processing Unit (GPU), is needed for the efficient parallel implementation of a deep neural architecture. For this purpose, a cloud-based communication has been established, between HoloLens and the computing machine containing GPU, using the Microsoft OneDrive (https://www.microsoft.com/en-ie/microsoft-365/onedrive/online-cloud-storage) synchronisation service. In particular, NVIDIA GeForce GTX 1060 (https://www.nvidia.com/en-gb/geforce/graphics-cards/geforce-gtx-1060/specifications/) GPU has been used to implement generative DNNs to enable the 3D model inference from a single-view 2D image. The DNNs have been trained using, a widely used 3D benchmark dataset called, ShapeNet [47]. The provided rendered images from 24 different angles for each 3D model make the dataset ideal for training DNNs to infer 3D models of real objects from the images taken from various directions independent of the background information. However, to further test our 3D model generation methodology on real-world images apart from the rendered images, we have used Pix3D [48] dataset which consists of real images captured in diverse environments. The following subsection describes the orchestration of the system’s constituents to enable 3D model design in MR for AM.

### System architecture

**Fig 3:**
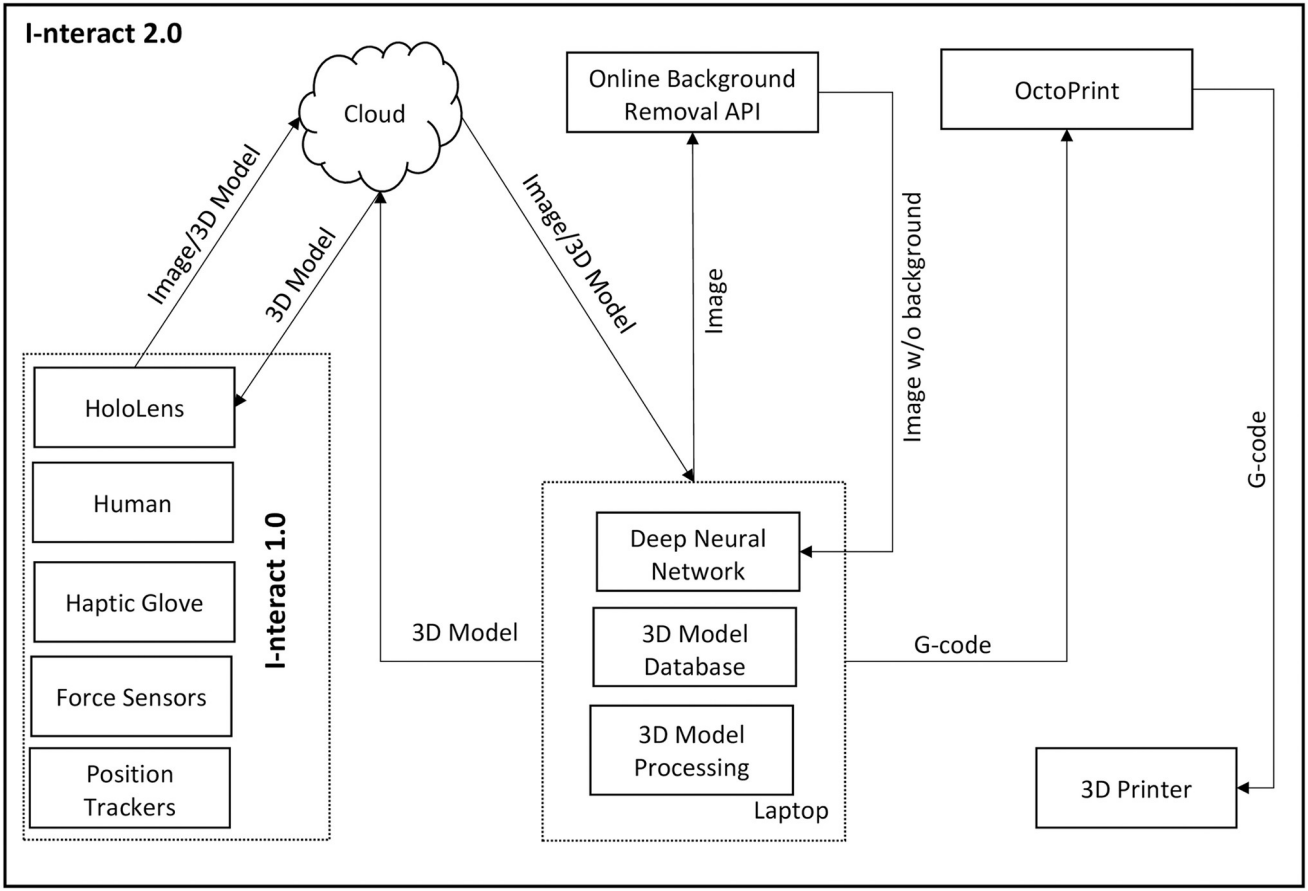
System architecture.

**Fig 4:**
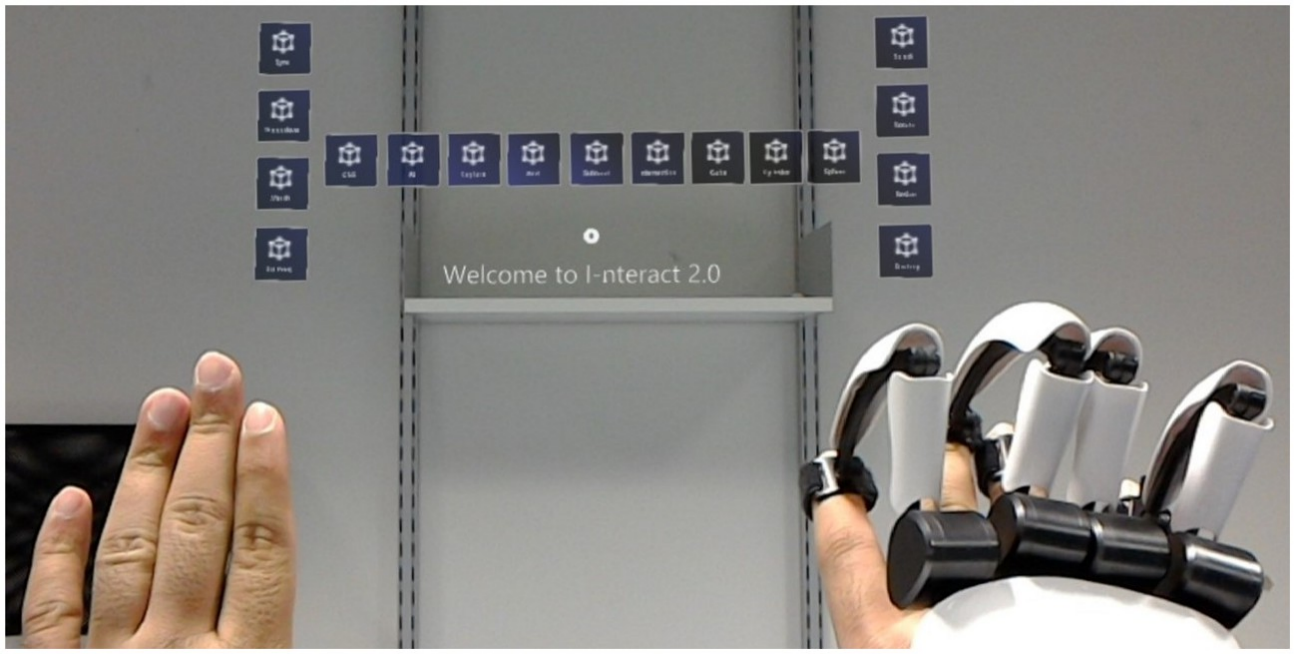
Interface for interaction.

The detailed system architecture that defines the flow of information between the different modules of I-nteract can be found in Fig 3 of [12]. The updated system architecture of I-nteract 2.0 after integration with CSG and DNN is depicted in Fig 3. As illustrated in Fig 3, the image or the 3D model is sent to the cloud to be accessed by the HoloLens and the computing machine (laptop). The 3D print controller application OctoPrint (https://octoprint.org/) has been used to send the 3D model to the printer PRUSA i3 MK3 (https://www.prusa3d.com/). The MR interface is shown in Fig 4. The interface consists of a hand with glove, a hand without glove, virtual buttons, and voice commands. The hand with glove can be used to translate, rotate, and resize the 3D model while getting force feedback. The hand without glove can be used to utilise the built-in interface of the HoloLens such as moving the 3D model and pressing the virtual buttons. The user can control the interface using voice commands, gestures (from the hand without haptic glove), and hand motions as well as finger motions (of the hand with haptic glove). The respective functions and the associated voice commands of the virtual buttons are detailed in Table 1.

**Table 1:** Virtual buttons, voice commands, and their respective functions.

Virtual Button / Voice Command	Function
Sync	To sync the position of the real hand with glove and the virtual hand model.
Cube	To start drawing a cuboid.
Sphere	To start drawing an ellipsoid.
Cylinder	To start drawing a cylinder.
Add	Union of the primitive shapes.
Subtract	Difference of the primitive shapes.
Intersect	Intersection of the primitive shapes.
Select	To select the 3D model.
Capture	To take an image using HoloLens.
Dimension	To display the dimensions of the selected 3D model.
Resize	To resize the selected 3D model.
Match	To find the best possible match of the selected 3D model from the database.
Print	To send the 3D model to the printer.

## Generating 3D models in a mixed reality environment

The conventional graphical user interface (GUI) for 3D modelling renders the virtual 3D world on a 2D computer screen. This makes the use of mouse and keyboard to locate and place virtual objects in a 3D environment unintuitive and difficult for inexperienced users. Also, most contemporary CAD-based software demands strong technical background which makes it even more difficult for non-technical consumers to participate in the design process [10]. In this context, there is a clear need for developing innovative interfaces that not only take advantage of MR technologies for interacting with 3D models in a three-dimensional environment but also enable generative CAD in MR and utilise ML to automate the parts of the design process that require expert knowledge. I-nteract is a CPS that provides a framework to develop such intuitive and automated interfaces for assembling, creating, interacting, modifying, positioning, and shaping 3D models within a three-dimensional environment. Built upon I-nteract, I-nteract 2.0 uses the generative functionalities of CSG and DL to enable the user to create 3D models from 3D primitive shapes as well as to automate the generation of the 3D models based on 2D images. Taking advantage of the immersive feature of MR, I-nteract 2.0 also allows the user to modify the dimensions of a 3D model with respect to the physical workspace.

### I-nteract to generate 3D models using constructive solid geometry

**Fig 5:**
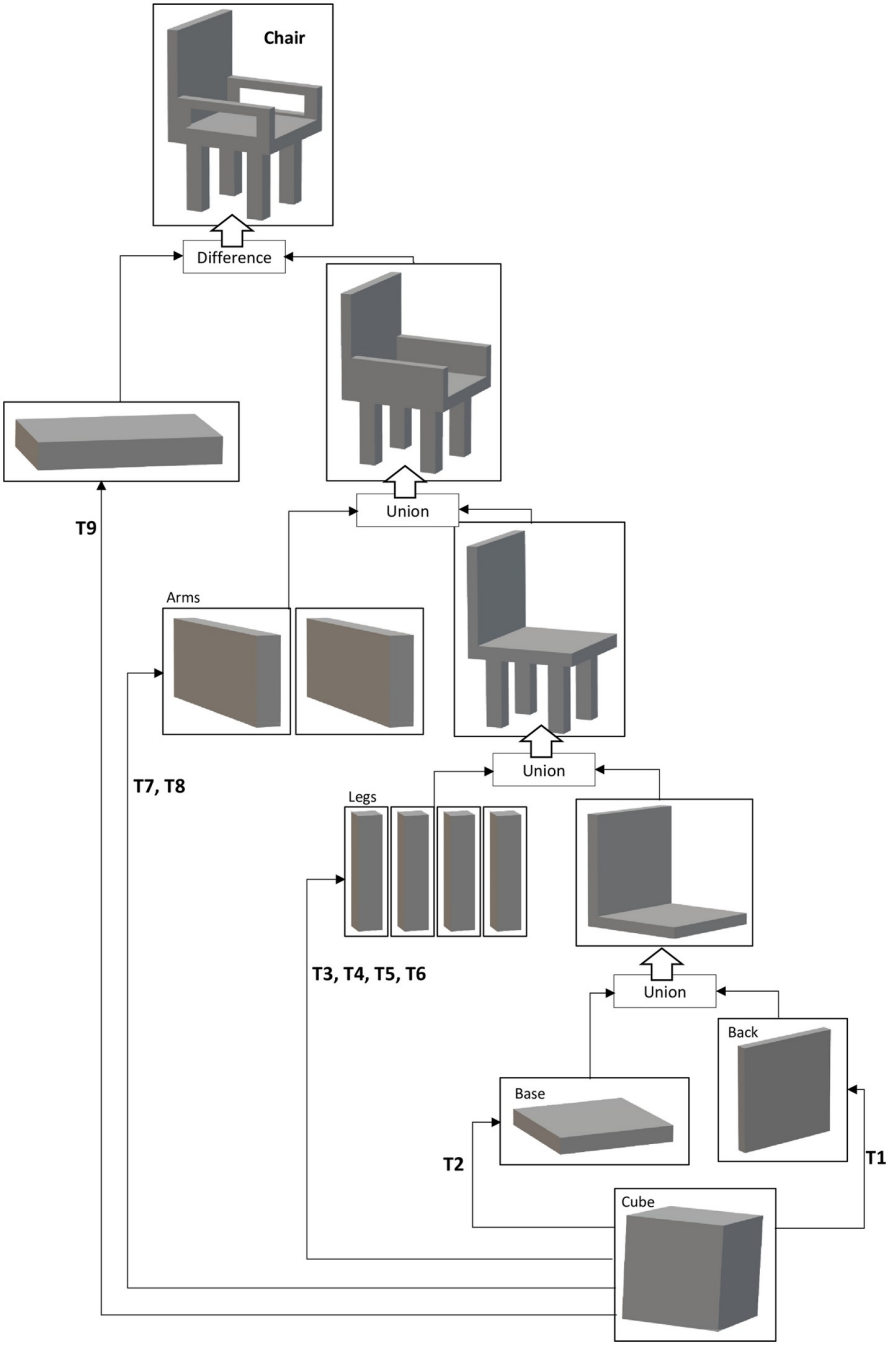
Generating 3D model of a chair using CSG.

**Fig 6:**
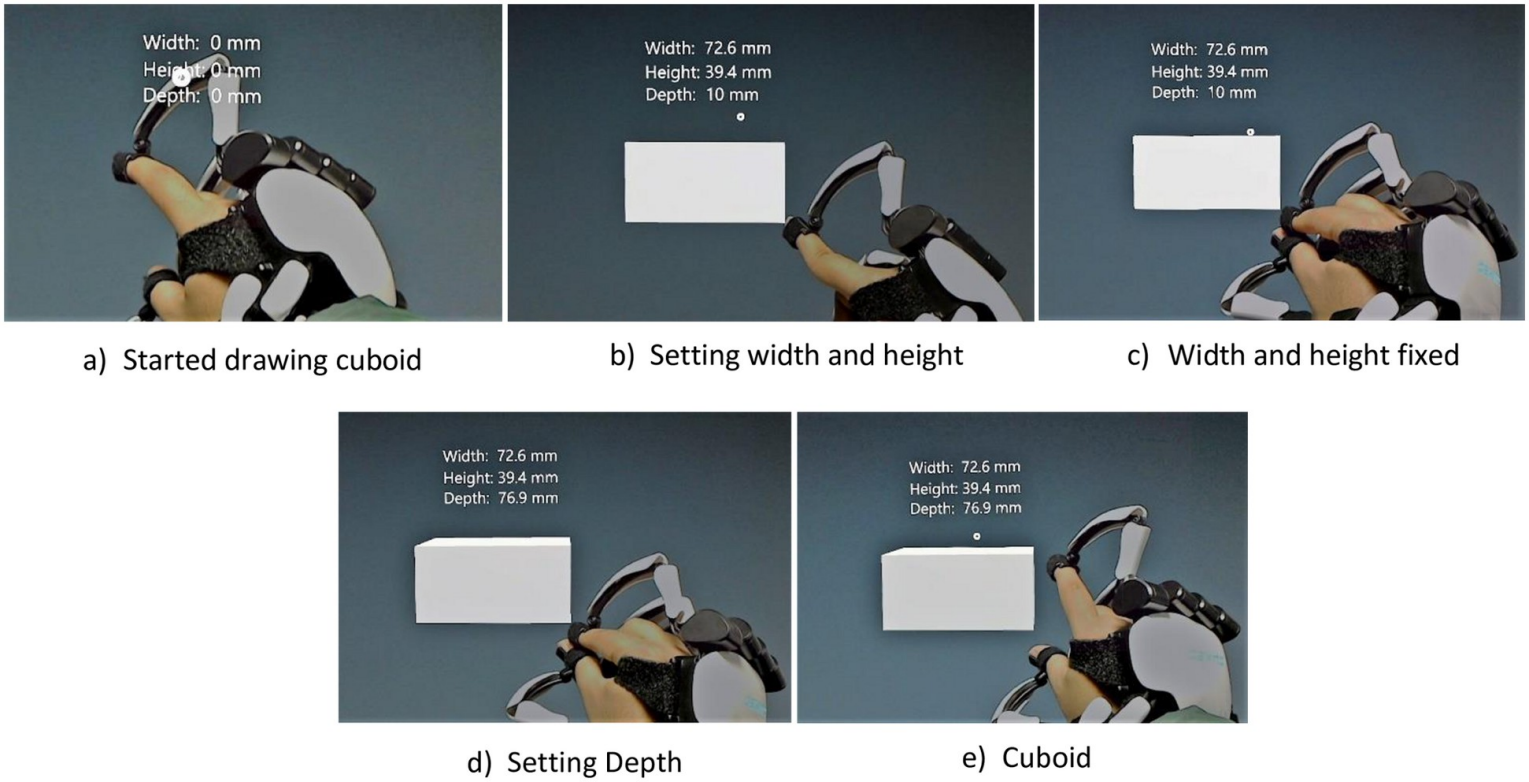
Drawing a cuboid in MR using I-nteract 2.0.

Constructive solid geometry (CSG), used in solid modelling, allows the user to construct complex 3D models by using boolean set operations (e.g., union, difference, and intersection) on simple building blocks (e.g., cubes, cylinders, and spheres) called primitives. CSG has been utilised in the system to enable the user to intuitively design 3D models in an MR environment from primitive shapes. An example of creating a chair using CSG is illustrated in Fig 5. Table 2 depicts the transformations applied to the cube in the example, shown in Fig 5, to translate, rotate, and resize the primitive shapes. The position, rotation, and scale vectors given in Table 2 are the same vectors that are used in Unity (https://unity.com/) to transform a 3D model. The hand with the glove can be used to grab (in order to translate or rotate the model) or resize the virtual object in the 3D physical workspace. The hand without the glove can be used to translate the virtual object. This feature is useful when the user is using the other hand (with glove) to resize the virtual object so that the user can place and resize/rotate the virtual object simultaneously in the physical workspace by using both hands. The procedure implemented to draw a 3D primitive shape using the hand with glove is illustrated by Fig 6(a)–6(e). The procedure implemented to generate a 3D model using CSG is described below (Video demonstration: https://youtu.be/KKf-q2r04TA).

**Fig 7:**
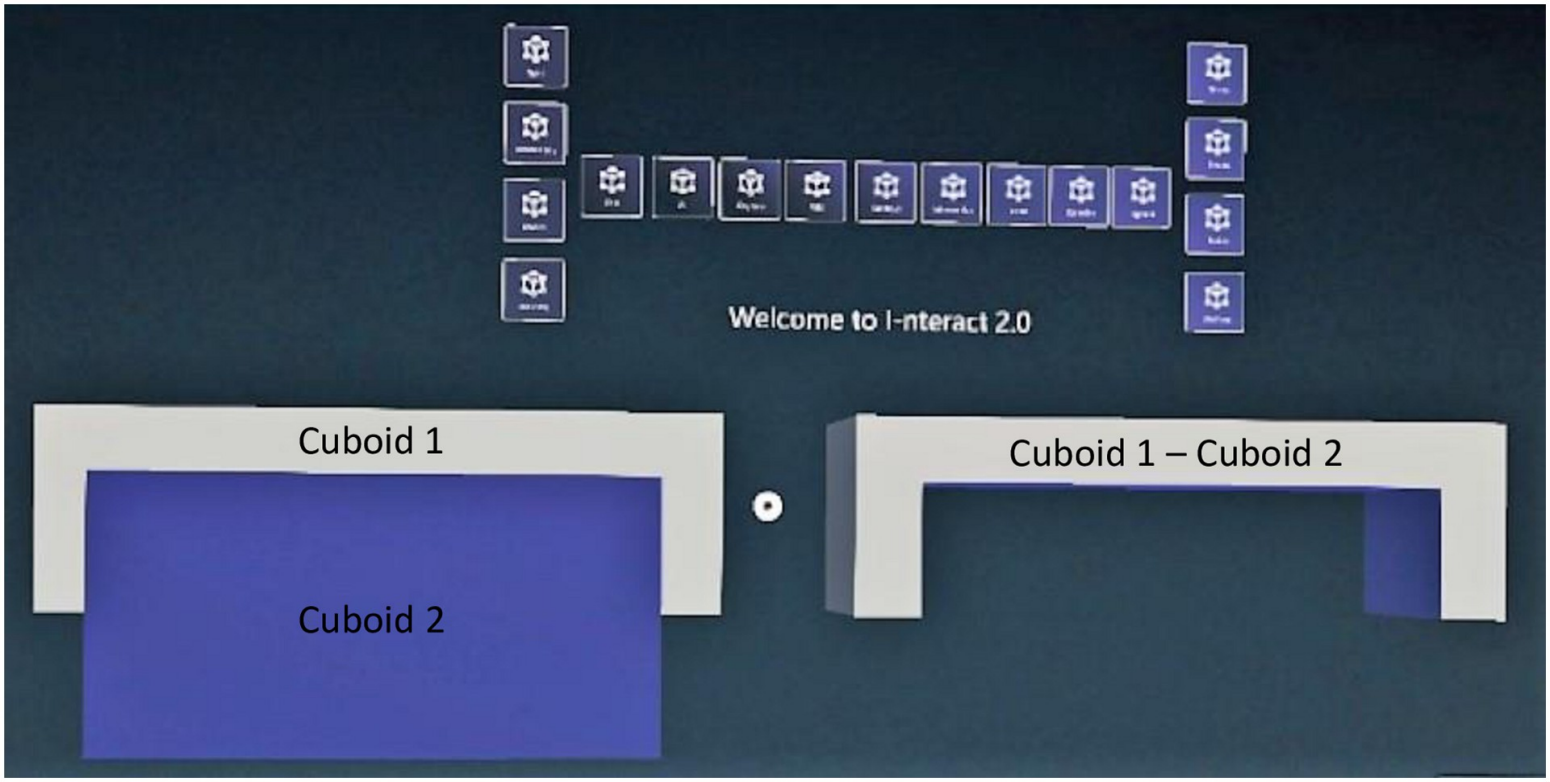
Subtracting two cuboids in MR using I-nteract 2.0.

1. Position the hand with glove in the physical workspace where the primitive shape is desired to be drawn. The index finger of the hand should be open as shown in Fig 6(a). Press the virtual button of the desired primitive shape (Cube, Sphere, Cylinder) using the hand without glove or use the associated voice command, as described in Table 1, to start drawing the primitive shape.
2. After selecting the desired primitive shape, the width and the height of the primitive shape can be adjusted by moving the hand with glove in left/right (x) and up/down (y) direction respectively as shown in Fig 6(b). Close the index finger of the hand when done as shown in Fig 6(c).
3. Move the hand in the forward/backward (z) direction to adjust the depth of the primitive shape as shown in Fig 6(d). Open the index finger of the hand with glove when done as shown in Fig 6(e).
4. Repeat steps 1 to 3 to draw another primitive shape.
5. Apply the transformations (translation or rotation) by grabbing the virtual object using the hand with glove to place the primitive shape at the desired location and orientation.
6. Press the “Select” virtual button or use the associated voice command and then press on the desired primitive shape to select.
7. After selecting the two virtual objects, press the virtual button of the desired boolean operation (Add, Subtract, Intersection) or use the associated voice command to apply the boolean operation (Union, Difference, Intersection respectively). Fig 7 shows the subtraction of two cuboids in MR using I-nteract 2.0.
8. Repeat steps 1 to 7 to generate a 3D model from the primitive shapes.

**Table 2:** Transformations to cuboid for CSG shown in Fig 5.

Transformations	Position Vectors	Rotation Vectors	Scale Vectors
T1	<0, 0.044, 0.4>	<0, 0, 0>	<0.1, 0.1, 0.01>
T2	<0, 0, 0.355>	<0, 0, 0>	<0.1, 0.012, 0.1>
T3	<-0.03, -0.034, 0.386>	<0, 0, 0>	<0.015, 0.07, 0.015>
T4	<-0.03, -0.034, 0.326>	<0, 0, 0>	<0.015, 0.07, 0.015>
T5	<0.03, -0.034, 0.326>	<0, 0, 0>	<0.015, 0.07, 0.015>
T6	<0.03, -0.034, 0.386>	<0, 0, 0>	<0.015, 0.07, 0.015>
T7	<-0.045, 0.017, 0.3525>	<0, 0, 0>	<0.01, 0.035, 0.095>
T8	<0.045, 0.017, 0.3525>	<0, 0, 0>	<0.01, 0.035, 0.095>
T9	<0, 0.0175, 0.3525>	<0, 0, 0>	<0.12, 0.018, 0.07>

S1 Video demonstrates a user generating a 3D model of a chair, using I-nteract 2.0, by following the above-mentioned procedure.

Generating complex 3D models from scratch could be a laborious task. The next section describes an automated approach to generate 3D models using DL for 3D printing.

### I-nteract to generate 3D models using deep learning

Taking advantage of the capability of DL to learn from complex high-dimensional data (such as images) and, hence, thereby to automate the generation of 3D models for novice users in MR the system has been integrated with DNN. To generate the 3D model of a physical object automatically using I-nteract, the user captures the image of a real object via HoloLens. The captured image is sent to the cloud to be accessed by the laptop as illustrated in Fig 3. As the DNN for the mesh generation is trained using the synthetic data of 2D images rendered from the 3D models, therefore, to use the DNN on the real images captured through HoloLens, the acquired image of the physical object needs to be preprocessed. To remove the background of the input image an online background removal API (https://www.remove.bg/) has been used. After removing the background, the image is cropped and resized to the image resolution of 64x64 to feed into the DNN. The 3D model generated from DNN is uploaded to the cloud to be accessed by the HoloLens and displayed to the user in MR. Two types of DNN architectures, Soft rasterizer (SR) [22] and SliceGen, for the single-view mesh reconstruction, have been implemented. The two generative DL frameworks are presented in the following subsections.

#### Generation by soft rasterizer

**Fig 8:**
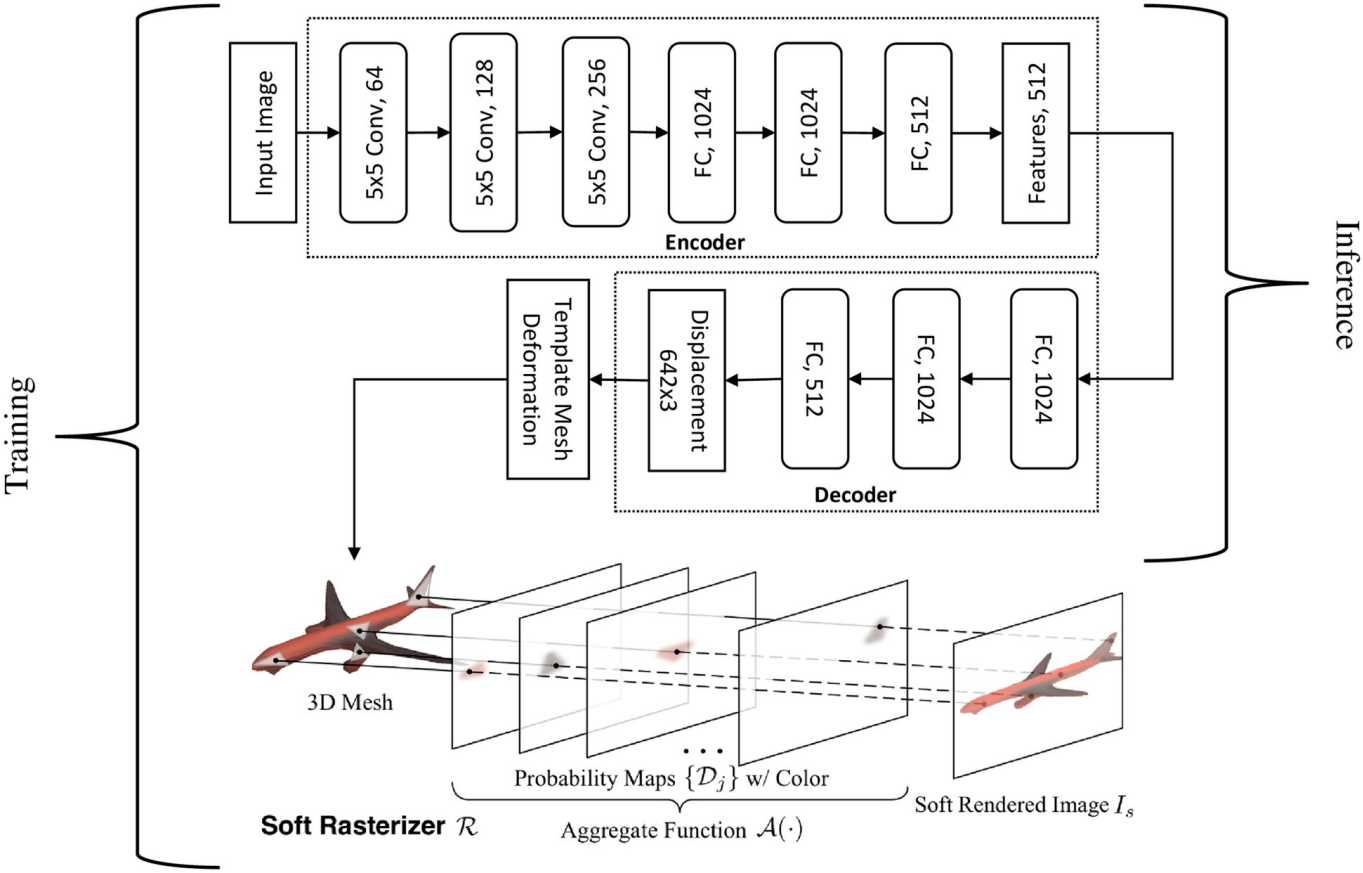
SR-DNN architecture.

**Fig 9:**
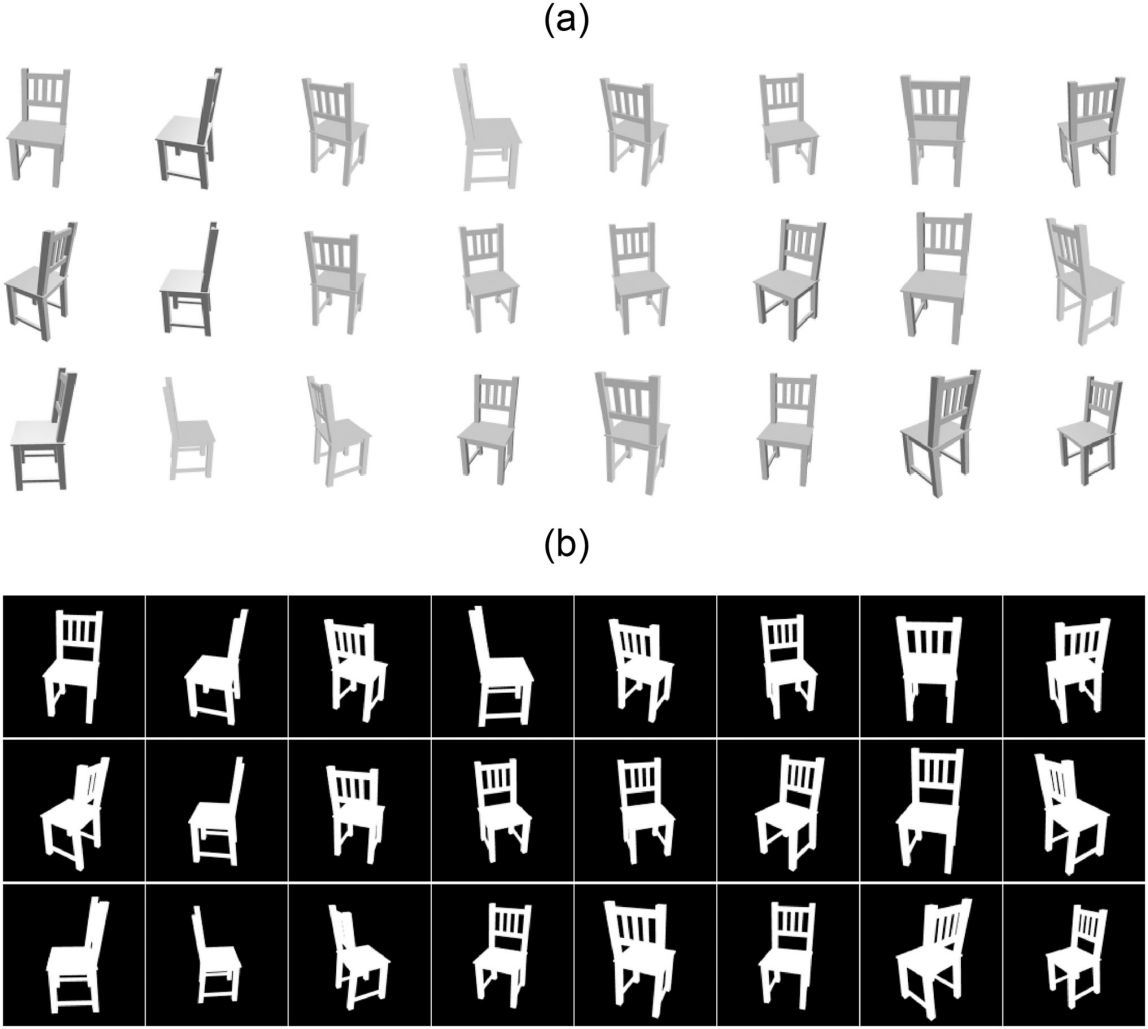
Training images of a 3D model for SR-DNN. A: Training input images for a 3D model with each image of resolution 64 × 64 × 4. B: Training output images for a 3D model with each image of resolution 64 × 64 × 1.

Rasterization is a widely used method within graphics pipelines to render 3D models on 2D screens [49]. The discrete sampling operations during rasterization make it non-differentiable and therefore unsuitable for the image-based 3D reasoning using DL as gradients are required for backpropagations to train the DNNs. SR [22] is a differentiable rendering framework to train a neural network to infer 3D information from 2D images. This learning approach combined with the encoder-decoder architecture [23, 24] can be used for mesh reconstruction of 3D models from a single view image by deforming a template mesh. An encoder-decoder architecture, identical to [22] for single-view mesh reconstruction, has been employed. The encoder is used as a feature extractor from the 2D images whereas the decoder generates the per-vertex displacement vectors that deform a template mesh (sphere) into a target model based on the input 2D image. The encoder contains three convolution (Conv) and three fully connected (FC) layers and outputs a feature vector. The decoder is composed of three FC layers and outputs per-vertex displacement vectors to deform a template mesh into the desired model. The detailed network structure is illustrated in Fig 8 [22]. The SR-DNN has been trained on a single NVIDIA GeForce GTX 1060 GPU and implemented using PyTorch. The dataset provided by [23], which contains 13 categories of objects from ShapeNet [47], has been used. Out of 13 categories, the DNN has been trained for two categories “Chairs” and “Tables”. Each 3D model is rendered in 24 different views with an image resolution of 64 × 64 and four channels to generate synthetic (2D images) data to train the DNN. Three channels of each image are RGB whereas the fourth one is its silhouette. The fourth channel (silhouette) of each input image is also used to compute loss for backpropagation during training. Fig 9A shows 24 different rendered views of a 3D model to be used as input images either during the training or the inference phase whereas Fig 9B depicts training (silhouette) images of the 3D model that are used to compute losses (supervision). During the training phase, the image batch (*B* × 64 × 64 × 4) with batch size *B* = 64 is fed into the encoder-decoder to obtain deformed meshes. The deformed meshes are then passed through the SR to generate silhouette images (*B* × 64 × 64 × 1). The generated silhouette images (${\hat{I}}_{s}$) are compared with output training (silhouette) images (*I*_*s*_) to compute IOU (silhouette) loss ($L_{s}$) for backpropagation using

$$\begin{array}{c} L_{s}=1-\frac{\|{\hat{I}}_{s}⊗I_{s}{\|}_{1}}{\|{\hat{I}}_{s}⊕I_{s}-{\hat{I}}_{s}⊗I_{s}{\|}_{1}} \end{array}$$ (1)

where ⊕ and ⊗ are the element-wise sums and products respectively and ‖ ⋅ ‖_1_ denotes *l*_1_-norm. The network has been optimised using Adam [50] optimisation algorithm with *α* = 10^−4^, *β*_1_ = 0.9, *β*_2_ = 0.999, and *ϵ* = 10^−8^. SR is used only during the training phase to generate a silhouette image of the mesh deformed by the encoder-decoder and is omitted after training during inference (single-view mesh reconstruction) as illustrated in Fig 8. The probability map and aggregate functions computations [22], involving exponential functions, for all the mesh template triangles times all the silhouette pixels make soft rasterization and hence the training process computationally expensive.

As mentioned above, SR-DNN reconstructs the mesh by deforming a template mesh (sphere) of genus zero, therefore all 3D models generated using SR-DNN are also of the same genus as that of the template mesh and hence is unable to match the topology of the real objects. In the subsequent section, a novel method of generating single-view image-based 3D models that does not limit the genus of the reconstructed mesh has been presented.

#### Generation by SliceGen

**Fig 10:**
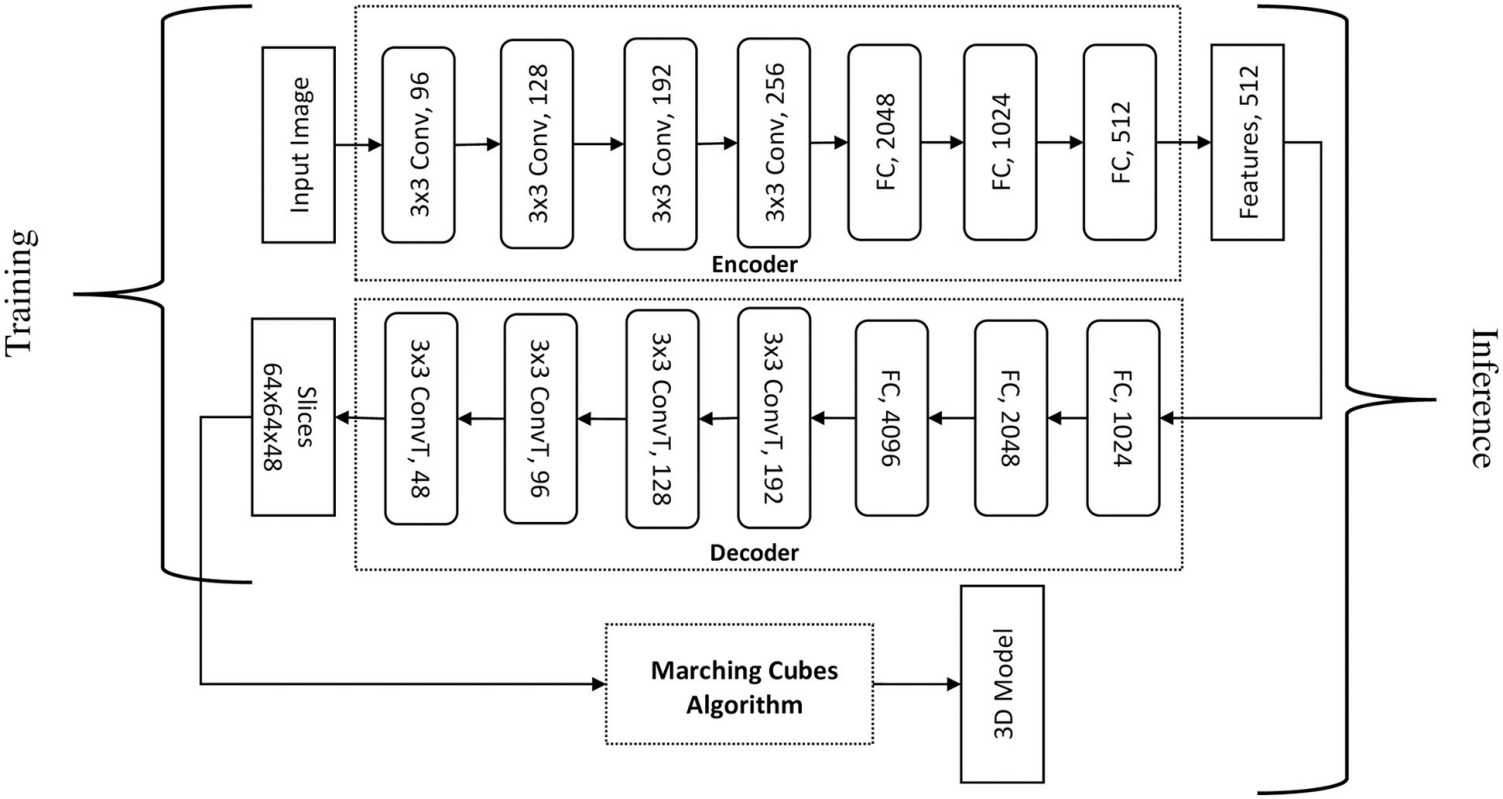
SliceGen architecture.

**Fig 11:**
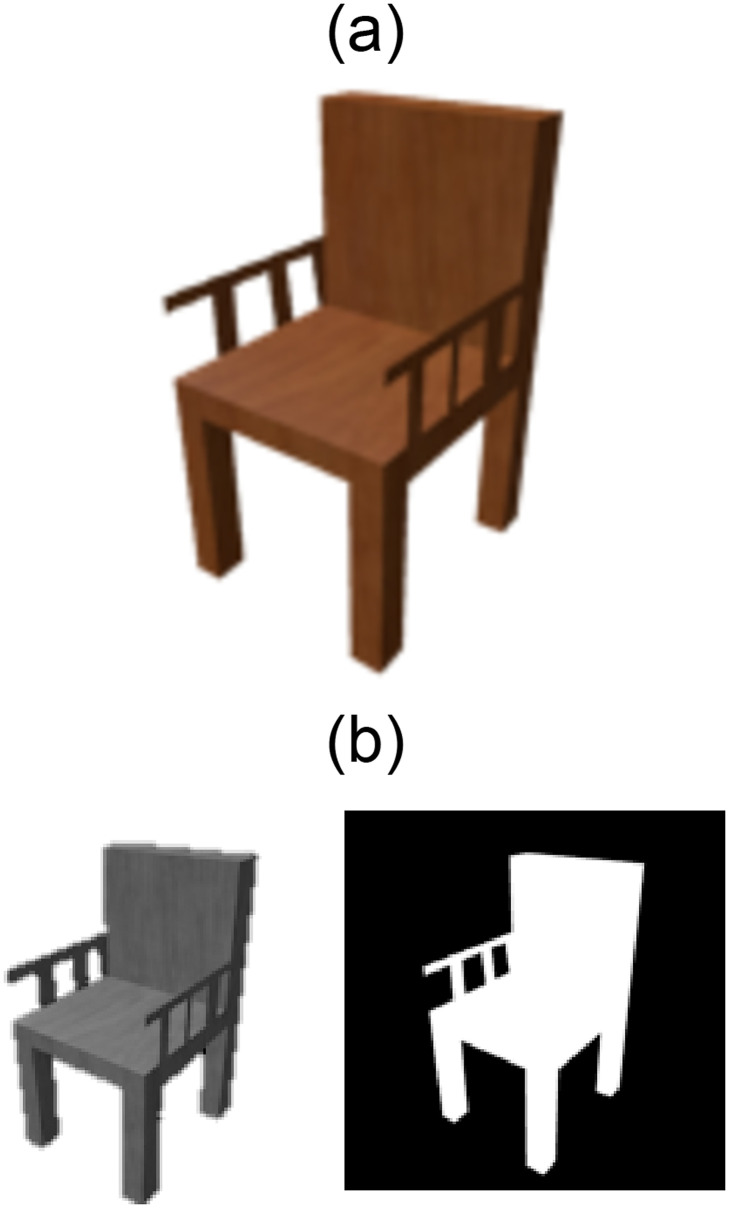
Conversion from 4 channels to 2 channels. A: Colored image from ShapeNet dataset (4 Channels). B: Processed image with 2 channels: grayscale (left) and silhouette (right).

Inspired from AM, in which physical objects are manufactured through layer-by-layer material deposition, SliceGen is a novel DNN that generates slices (layers) of a 3D model based on a single-view 2D image of the target object. An encoder-decoder architecture similar to that presented in the previous section has been employed but instead of deforming a template mesh, SliceGen generates slices (layers) of the target model. These slices are then stitched together into a 3D model of the target object using an isosurface extraction technique called marching cubes [51]. The detailed architecture of the proposed DNN is illustrated in Fig 10. The encoder is used as a feature extractor from 2D images whereas the decoder generates slices of the target model. The encoder consists of four Conv and three FC layers to output a feature vector and the decoder contains three FC and four transposed convolution (ConvT) layers to generate 48 slices of the target 3D model. The proposed DNN has been trained on a single NVIDIA GeForce GTX 1060 GPU and implemented using TensorFlow. ShapeNet dataset [47] has been modified to train our DNN for two categories “Chairs” and “Tables”. The dataset comprises images of 24 different rendered views for each 3D model. Each image in the ShapeNet dataset consists of four channels (RGB + silhouette). As we are interested in only mesh reconstruction of the target 3D models and not in the inference of texture from the 2D images, therefore, to reduce the number of parameters to be trained in the input layer all the ShapeNet dataset images of the selected categories (chairs and tables) have been processed to contain two channels (grayscale and silhouette) as shown in Fig 11. The slices of the 3D models of the two categories in the ShapeNet dataset have been generated to be used as training output images for loss computations (supervision). The steps for generating 2D slices (layered images) of 3D models are as follows.

1. Convert the OBJ file format to STL using Blender 2.79 (https://www.blender.org/download/releases/2-79/).
2. Repair the STL files using an automated tool provided by Netfabb (https://www.autodesk.com/products/netfabb/overview).
3. Generate G-codes for the repaired STL files using Slic3r 1.3.0 (https://slic3r.org/releases/1.3.0/).
4. Generate the slices (layered 2D images) from the G-code files using a G-code simulator.

**Fig 12:**
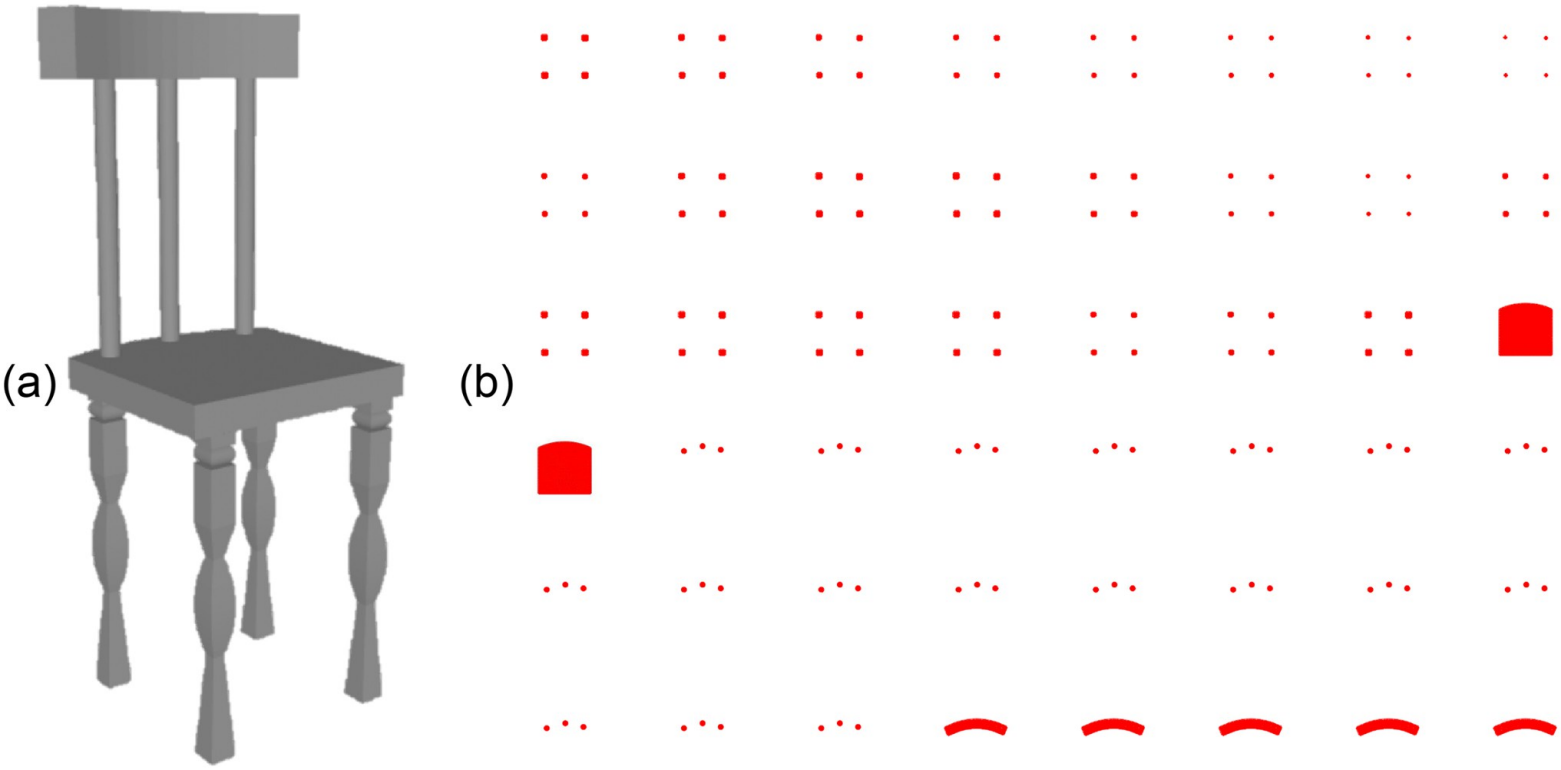
3D model to slices (RGB images of 3D model layers). A: 3D model from ShapeNet Dataset. B: Slices of the 3D model (64 × 64 × 3 × 48).

**Fig 13:**
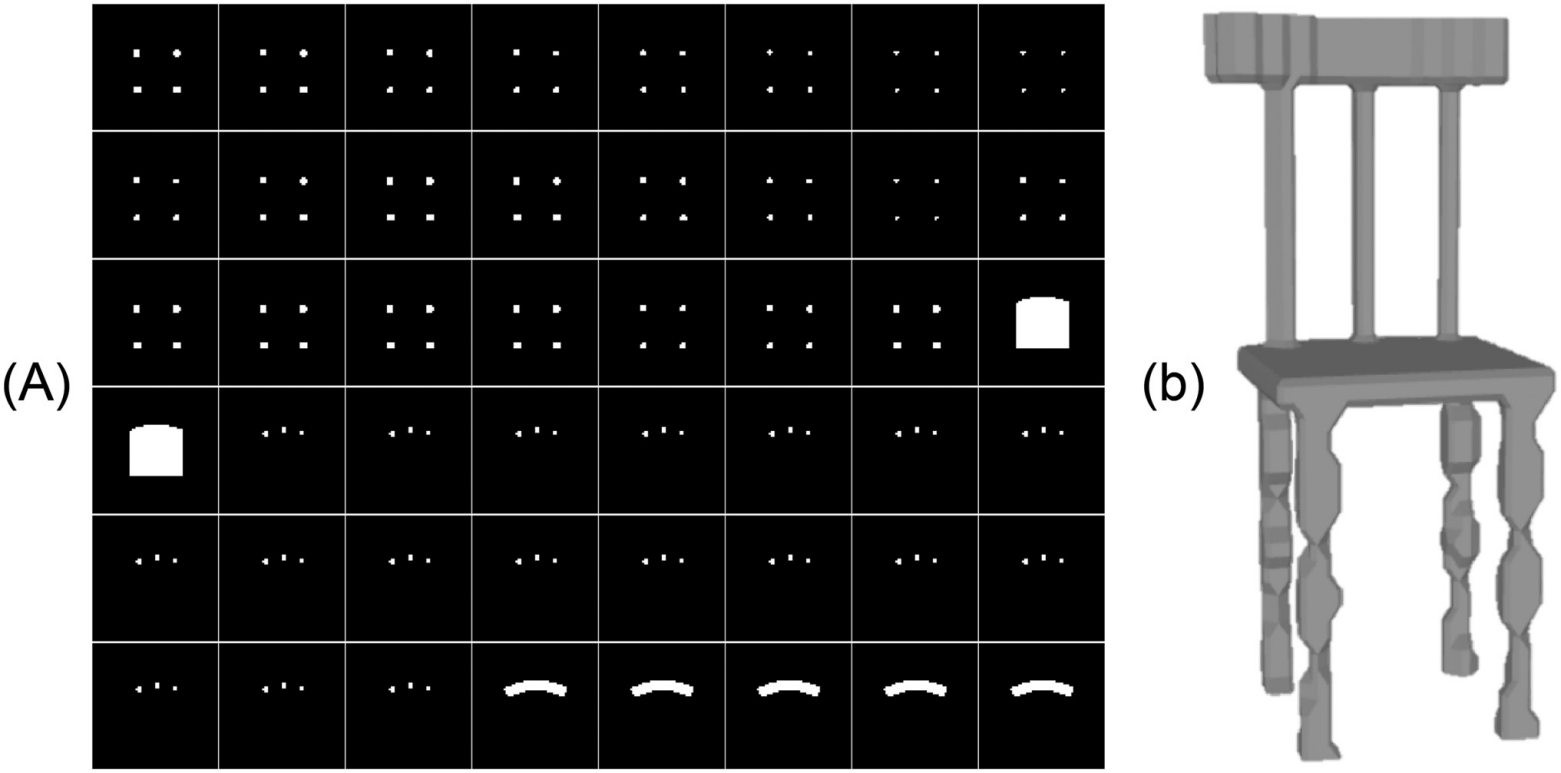
3D model reconstruction using marching cubes algorithm. A: Binary Slices (64 × 64× 1 × 48). B: Reconstructed 3D model from binary slices.

ShapeNet dataset has 3D models in OBJ file format, therefore, they are required to be converted to STL files as G-code generators (such as Slic3r) need 3D models to be in STL file format to create G-code files. Also, 3D models in the ShapeNet dataset are not 3D printable and hence are needed to be repaired to generate G-code files. Netfabb has been used to repair the ShapeNet dataset 3D models. The G-code simulator to generate slices from the G-code files has been adapted from [52]. Fig 12B depicts RGB images of the layers of the 3D model shown in Fig 12A. These slices are further processed to binary images, referred to as binary slices (*I*_*s*_), as shown in Fig 13A. The binary slices of each 3D model are used as training output images to compute backpropagation losses.

During the training phase, the image batch (*B* × 64 × 64 × 2) with batch size *B* = 64 is fed into the encoder-decoder to generate binary slices (*B* × 64 × 64 × 48). The generated binary slices (${\hat{I}}_{s}$) are compared with output training binary slices (*I*_*s*_) to compute IOU loss ($L_{s}$), similar to Eq 1, for backpropagation using

$$\begin{array}{c} L_{s}=1-\frac{\|{\hat{I}}_{s}⊗I_{s}{\|}_{1}}{\|{\hat{I}}_{s}⊕I_{s}-{\hat{I}}_{s}⊗I_{s}{\|}_{1}} \end{array}$$ (2)

where ⊕ and ⊗ are the element-wise sums and products respectively and ‖ ⋅ ‖_1_ denotes *l*_1_-norm. The network has been optimised using Adam [50] with *α* = 10^−3^, *β*_1_ = 0.9, *β*_2_ = 0.999, and *ϵ* = 10^−8^. The marching cubes algorithm to reconstruct the 3D model from binary slices has been adapted from [53]. Fig 13B shows the 3D model reconstructed from binary slices depicted in Fig 13A using the marching cubes algorithm [51].

After the automated generation of the 3D models using the integrated DNN (either SR-DNN or SliceGen) the user then can resize the generated 3D model to fit the dimensional constraints imposed by the physical workspace in MR. The resizing of the 3D model using I-nteract is described in the subsequent section.

### Resizing 3D models in the physical workspace

I-nteract 2.0 provides an intuitive interface to resize a 3D model using hand motion in an MR environment. This functionality can be used to resize a 3D model according to the space in the real world. The procedure implemented to resize a 3D model using I-nteract 2.0 is described below (Video demonstration: https://youtu.be/MwYldR-1OCM).

1. Press the “Select” virtual button using the hand without glove or use the voice command “Select” and then press on the 3D model (like pressing any virtual button) to select the 3D model.
2. After selecting the desired 3D model, press the “Resize” virtual button or use voice command “Resize”. The index finger of the hand with glove should be open while resizing the 3D model. The width, height, and depth of the 3D model can be adjusted by moving the hand with glove in left/right (x), up/down (y), and forward/backward (z) direction respectively. Close the index finger of the hand with glove when done.

**Fig 14:**
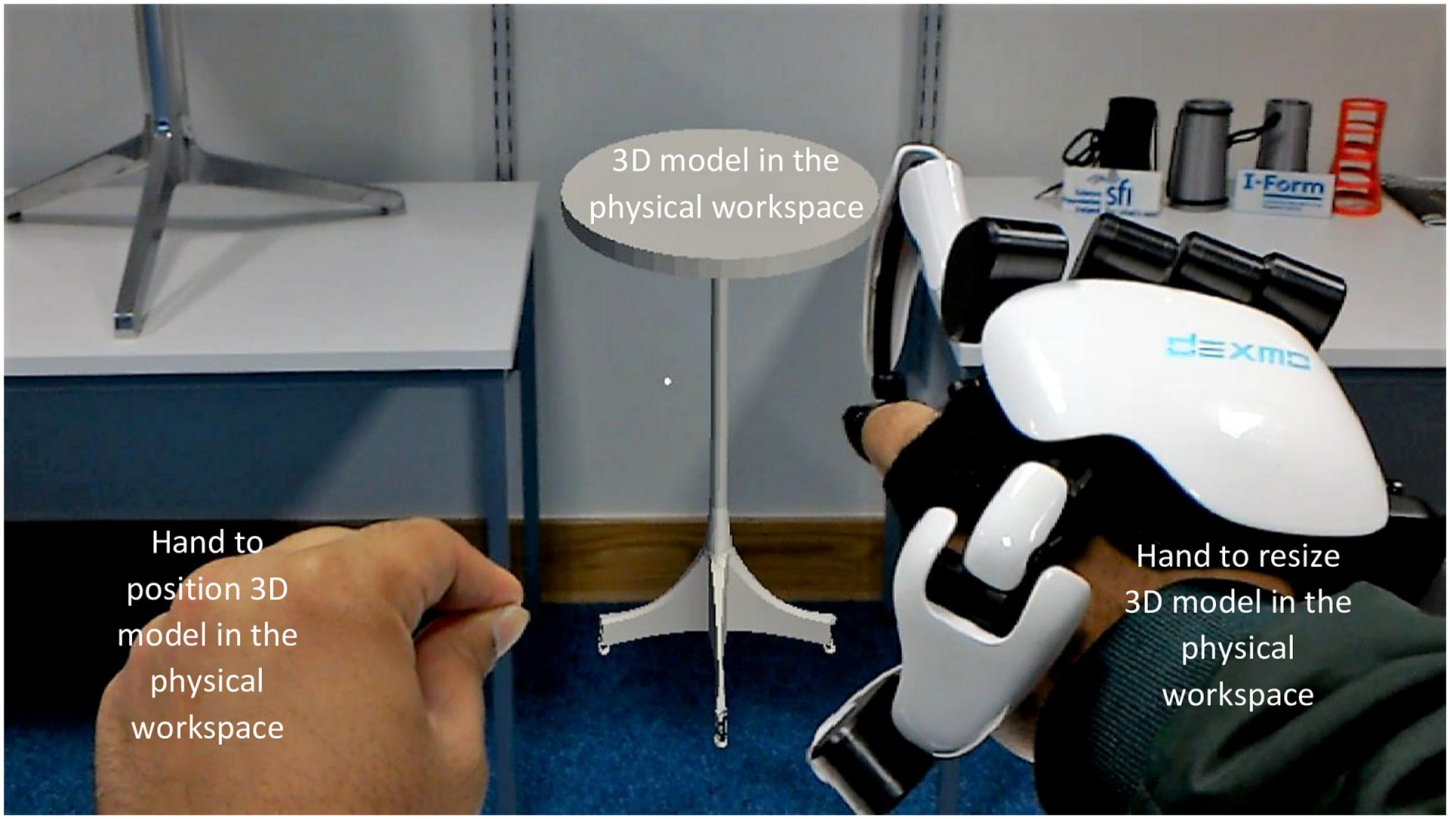
Resizing 3D model to fit in the physical workspace using I-nteract 2.0.

On the execution of the “Resize” command, the HoloLens records the position of the hand with glove. The HoloLens then updates (scales) the x, y and z-coordinates of the vertices of the 3D model with respect to the change in the hand position in x (left/right), y (up/down), and z (forward/backward) direction respectively. As the hand with glove will be in use while resizing the 3D model, therefore the user can use the hand without glove to position the 3D model in the physical workspace via the built-in gesture (and “ManipulationHandler” script) of the HoloLens as shown in Fig 14. S2 Video demonstrates a user resizing the 3D model of a table to fit in the physical workspace using I-nteract 2.0.

## Results and discussion

In this section, results have been demonstrated and the future developments in the generative functionalities of I-nteract 2.0 have been discussed.

### I-nteract to design customized 3D models in MR

**Fig 15:**
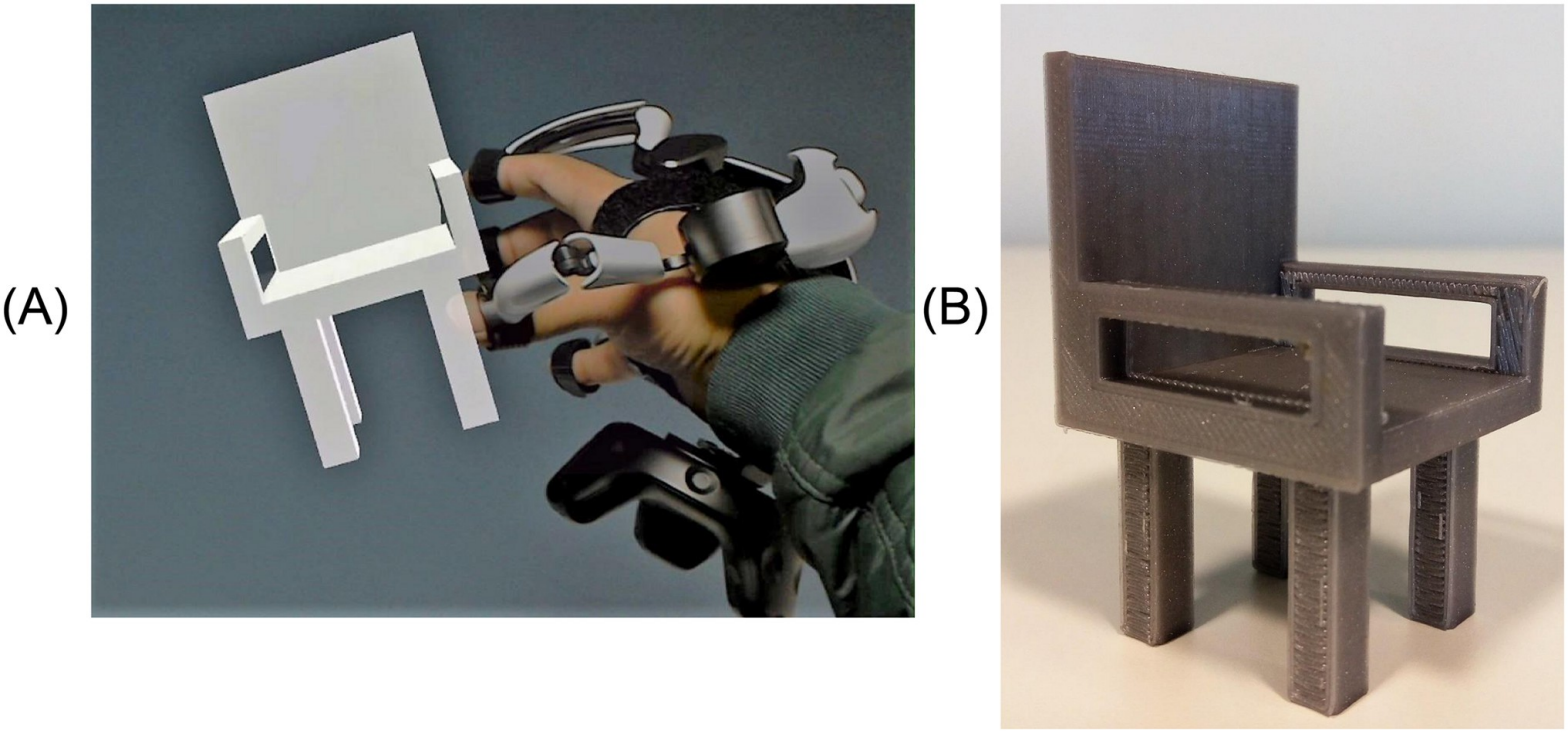
CSG generated chair using I-nteract. A: User interacting with CSG generated chair in MR. B: 3D print of a chair generated using CSG.

Fig 15A depicts a user interacting (translating, rotating, and getting force feedback) with the CSG generated 3D model of a chair in MR using I-nteract. The 3D model has been generated by applying transformations and boolean operations to primitive shapes (cube) as illustrated in Fig 5 in an immersive MR environment using I-nteract. The 3D print of the generated 3D model is shown in Fig 15B.

For quantitative comparison of the generative performance of the proposed SliceGen DNN architecture, a standard reconstruction metric, 3D intersection over union (IoU) [22], was used on the test dataset of ShapeNet over the chair and table categories. The mean IoU scores are presented in Table 3. The relatively higher mean IoU score by SliceGen depicts better performance due to the added degree of freedom of generating objects with multiple genera.

**Table 3:** Comparison of mean 3D IoU score with the baseline reconstruction methods on two categories of ShapeNet datasets.

Method	Chair	Table
**NMR [23]**	0.4990	0.4829
**SoftRas [22]**	0.5470	0.5325
**SliceGen**	0.645	0.586

**Fig 16:**
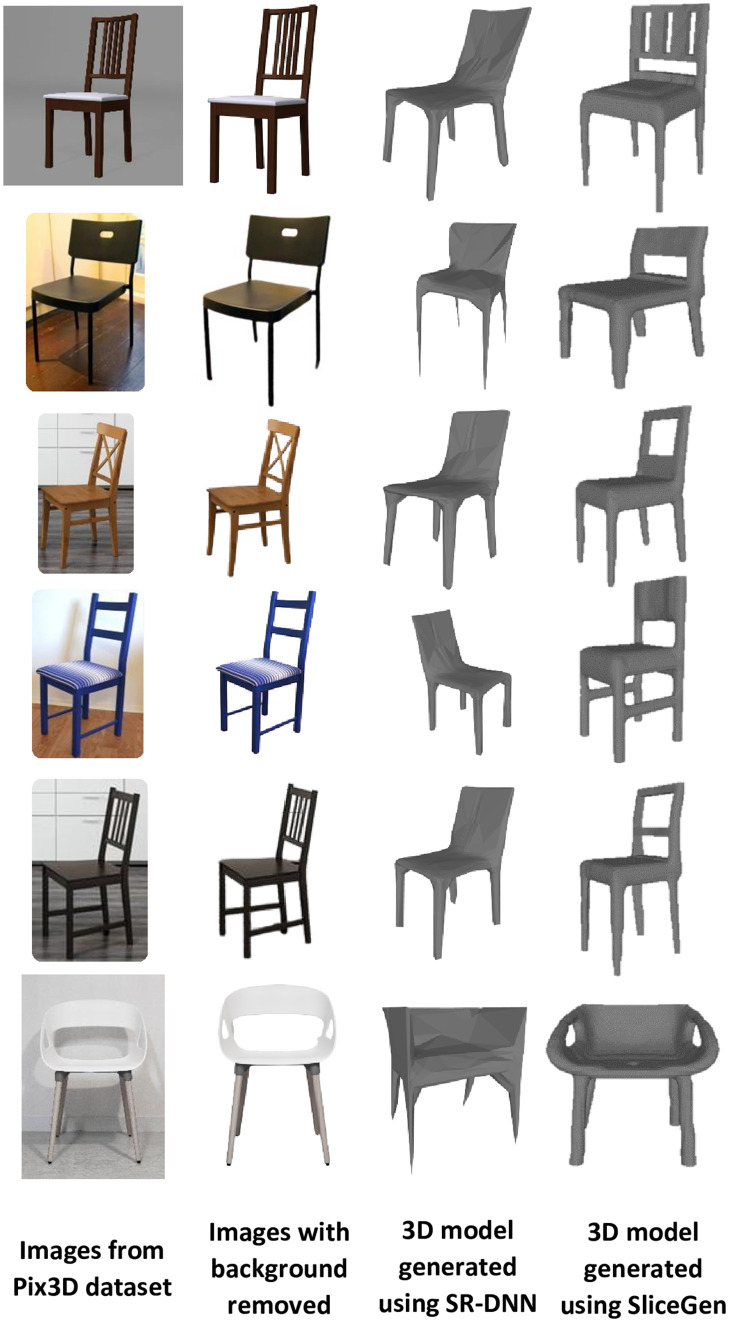
SR-DNN and SliceGen tested on real images from Pix3D dataset.

To test SR-DNN and SliceGen on real images to generate 3D models, firstly, Pix3D [48] dataset has been used. Pix3D is a dataset that consists of real images captured in diverse environments and ground-truth 3D models with nine object categories. Both integrated DNNs have been tested on the chair dataset and the corresponding results are shown in Fig 16. It can be observed in Fig 16 that SliceGen is able to generate 3D models with different genera whereas SR-DNN is generating 3D models only of genus zero. As both DNNs are trained using the synthetic data consisting of images without any background, noise, and occlusion with multiple views rendered from the 3D model of an object, therefore, are not robust and require a noise-free, transparent background image with a complete 2D view of the object to perform mesh reconstruction. That is why, images from the dataset which are clear, consist of a simple background, and are without any occlusion have been tested for single-view mesh reconstruction using the integrated DNNs. Future developments of our system will be devoted to training the DNNs on challenging and realistic datasets like Pix3D [48]. This will improve the robustness of the DNNs to extract features directly from the pictures and hence making the use of AI-based background removal API (https://www.remove.bg/) redundant.

**Fig 17:**
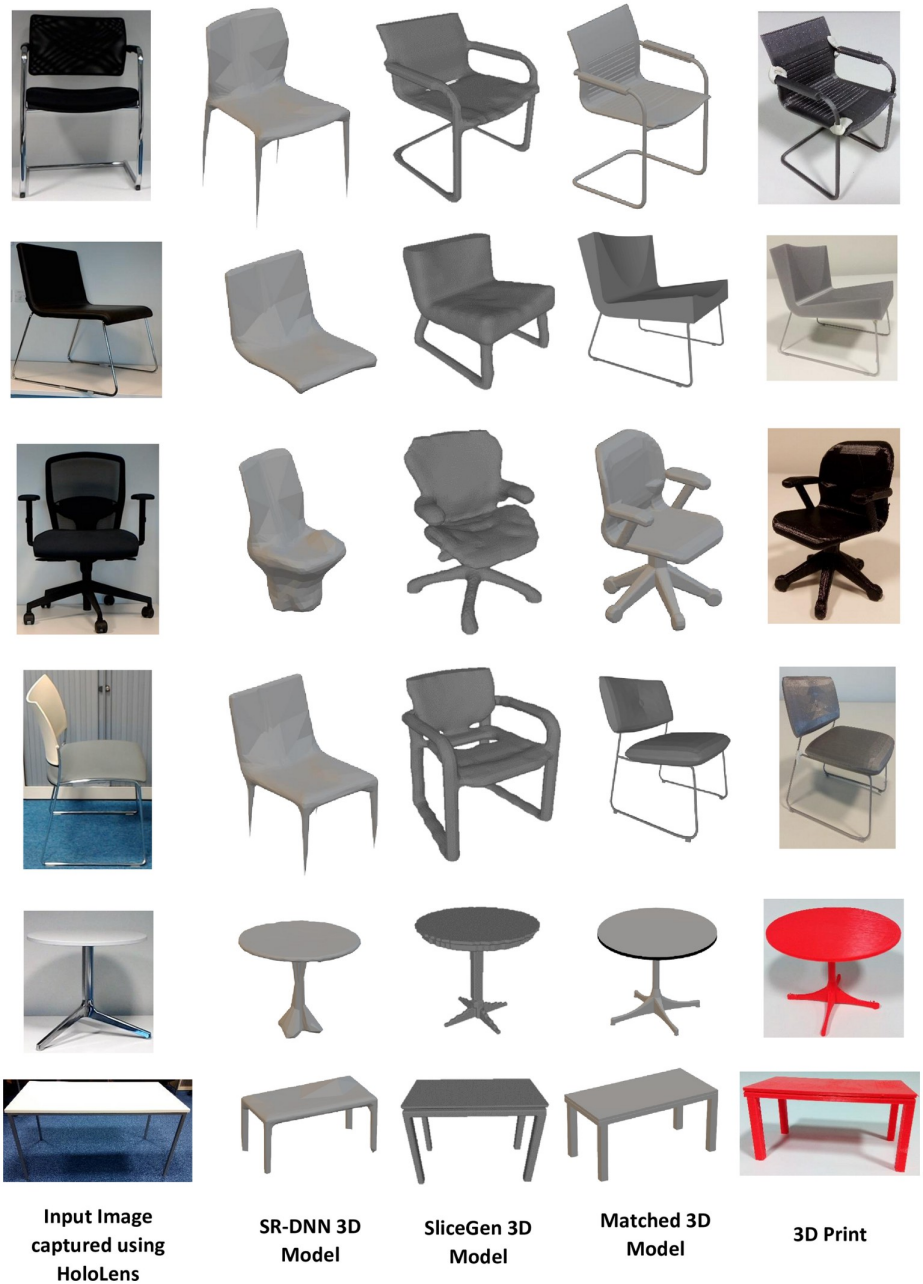
Images, the corresponding 3D models, and 3D prints.

After successfully testing DNNs on the Pix3D dataset, both SR-DNN and SliceGen integrated with the system (I-nteract) were tested for 3D model generation based on a 2D image. The images captured using HoloLens, the images after removing the background, the 3D models generated by SR-DNN, and by SliceGen are depicted in the first three columns of Fig 17. The time taken by the system from capturing a 2D image to the 3D model generation and presenting it to the user in MR has been recorded on average to be 20 seconds for both DNNs. Hence, provides a significant advantage as compared to generating a desired 3D model using a CAD design tool without any technical knowledge or training.

**Fig 18:**
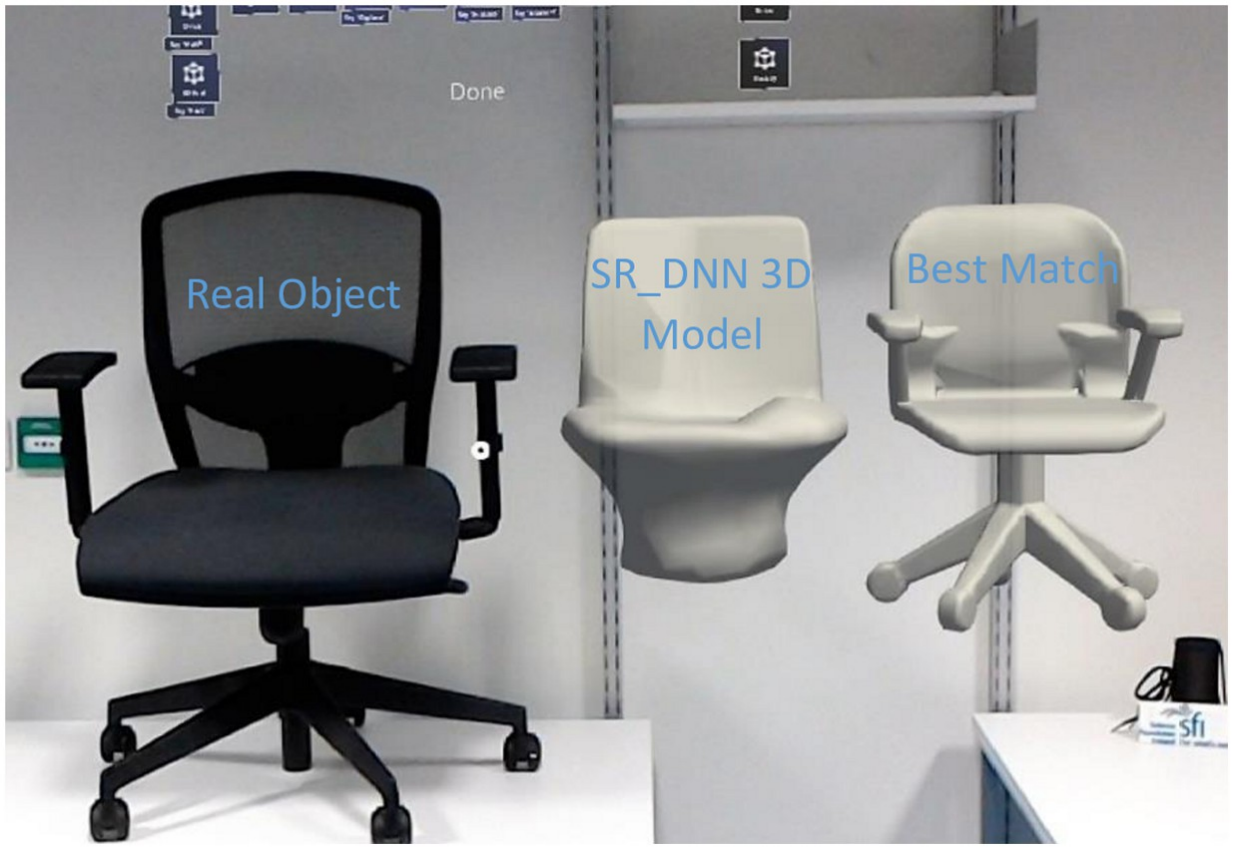
Real object, SR-DNN generated 3D model and the best match in MR.

Although an active research area, the single view 3D mesh reconstruction [22, 54–61] is still in its infancy in the context of being able to generate 3D models for AM. Contemporary generative DNN architectures are only focused on the 3D shape inference from 2D images while overlooking the mechanical design constraints, such as impact strength, tensile strength, flexural, and compression strength etc, in the supervised learning. Therefore, the generated 3D models by generative DNNs are not suited for 3D printing. For this reason, the 3D IOU metric [22] has been used to find the best match of the reconstructed mesh from a 3D model database that can be 3D printed. The HoloLens sends the generated 3D model to the cloud to be accessed by the laptop. The laptop then computes the 3D IOU score of the generated 3D model with all the 3D models in the database. The 3D model in the database with the maximum score (best match) is then sent to the cloud to be first accessed by the HoloLens and then displayed by the glasses to the user in MR. As an illustrative example, Fig 18 depicts a chair, a 3D model generated using SR-DNN, and the best match shown to the user in MR using I-nteract. The model with the highest 3D IOU score is the best quantitative match but might not be the best qualitative match from the user’s perspective [48]. Therefore generated 3D models with the top five scores are displayed to the user for qualitative assessment. The user can choose the best qualitative match for 3D printing. The 3D IOU matched 3D models from the database (ShapeNet dataset) and their 3D prints are depicted in the fourth and fifth columns of Fig 17 respectively.

**Fig 19:**
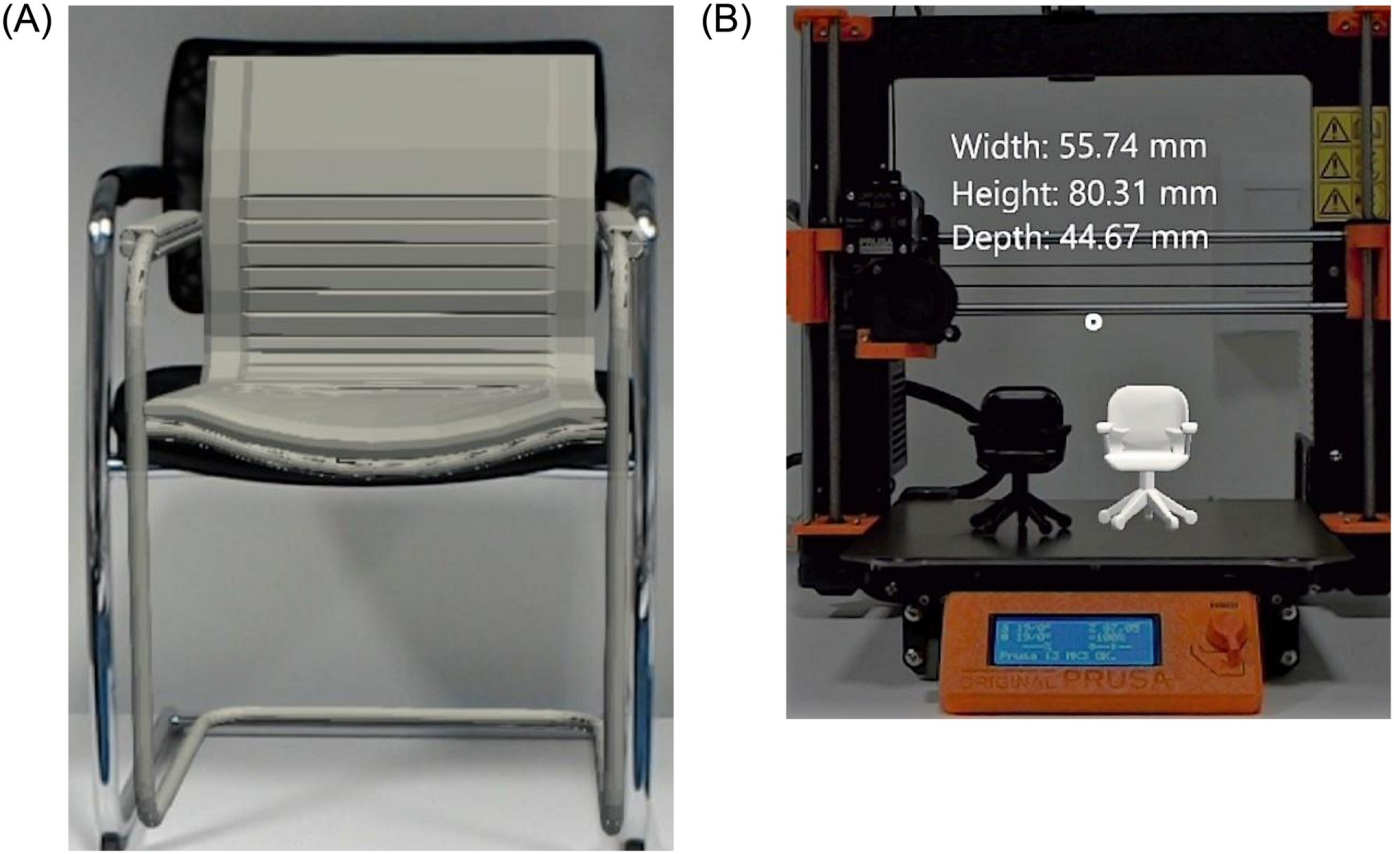
Resizing 3D models w.r.t physical workspace using I-nteract 2.0. A: Resizing the 3D model of a chair w.r.t a physical chair. B: Resized 3D model and its 3D print.

The 3D models resized with respect to the dimensional constraints imposed by the physical workspace using I-nteract are shown in Figs 14 and 19. Fig 19A shows the user resizing the matched 3D model of the chair by projecting the 3D model onto the real chair. Fig 14 depicts the user resizing the 3D model of a table to fit in a physical workspace between the two real tables. Fig 19B shows the resized 3D model of a table onto the base plate of the 3D printer along with its 3D print. To view the dimensions of a 3D model while resizing as shown in Fig 19B the user can execute the dimension command either via the virtual button or voice. The dimensions are computed based on the vertices positions in the OBJ file, allowing to display the maximum width, height, and depth of the 3D model. The maximum deviation between the resized dimensions and the measured dimensions of all the 3D-printed objects was recorded to be |2.1| mm which is highly dependent on the tracking accuracy of the HTC Vive trackers (https://www.vive.com/ca/vive-tracker/) used for hand tracking.

**Fig 20:**
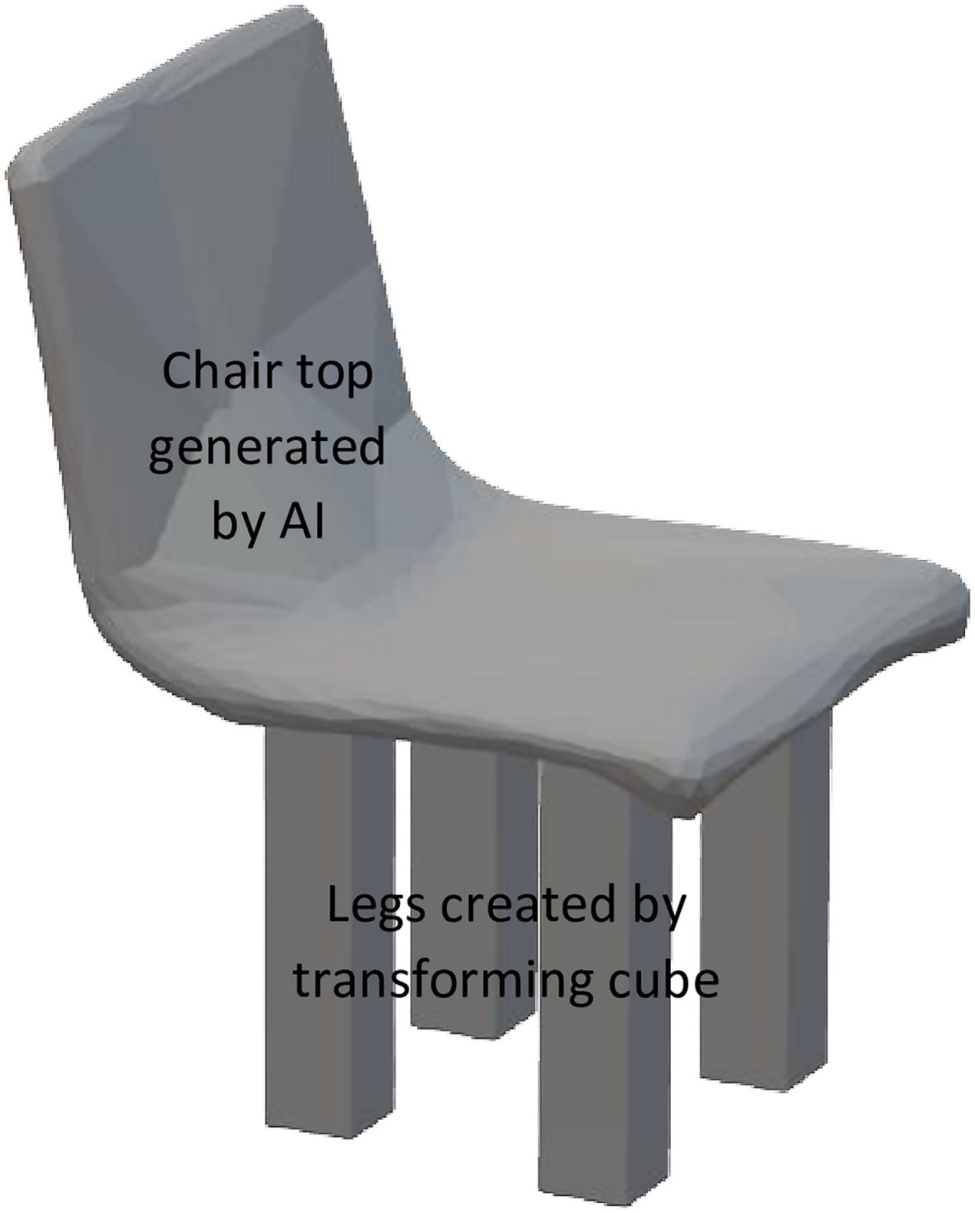
Modified DNN generated 3D model using CSG.

Future work includes enabling the user to modify the DNN generated 3D model using CSG in MR such as illustrated in Fig 20. In Fig 20, it can be seen that the 3D model generated from the image of a chair by SR-DNN does not have legs. Hence the 3D model is modified by adding legs using CSG. The modified model shown in Fig 20 has been created using Blender (https://www.blender.org/). This kind of interface will allow the user to easily modify an existing 3D model without the need to create a 3D model from scratch. The user can capture an image of the desired object or download it from the internet to get the 3D model from the DNN and further modify it using CSG. The user can also modify an existing 3D model downloaded from the internet using CSG. The metric for finding the best match from the database (like 3D IOU) will make sure that the modified model can be 3D printed or a CAD repair API (e.g., Netfabb—https://www.autodesk.com/products/netfabb/overview) can be integrated with the MR system to make the modified 3D model printable. Another interesting application that emerges from using generative DNN is the latent space interpolation and arithmetic [29]. Enabling latent space interpolation in I-nteract will allow the user to take images of two objects and generate a 3D model based on the objects in the two images. Future work will also be devoted to the use of haptic force feedback and force sensing capabilities of I-nteract to enable the user to transform the shape of a virtual object using hands in an MR environment. Mass customization enables customers’ participation in the creation process to integrate their input at a mass-production scale. This can be achieved by having multiple custom features that can be added by the customer, or by using AI to automate the design process as presented in this paper. The facilitation of product customisation as per the client’s need provide a competitive advantage to earn customer loyalty and build brand awareness, especially in the manufacturing sector [62]. Industries have already started to adopt this prosumer-based [3] manufacturing approach. Riddell, a sports equipment manufacturer, and Carbon, a 3D-printing company, are producing customised 3D-printed helmets for better protection to prevent head injuries (https://www.carbon3d.com/news/press-releases/riddell-carbon-produce-football-helmet). Similarly, Nike (https://www.nike.com), BMW (https://www.voxelmatters.com/bmw-ends-mini-yours-customised-service/), and many other industries are providing services to facilitate customisation using modern technological advancements. Using the framework presented in this paper for the integration of computationally expensive generative DNNs, future works also involve the implementation of reinforcement learning or natural language processing to take advantage of the customizability and flexibility of the AM process by enhancing the human-machine interaction such as the development of an AI-based human (consumer, designer, or operator) support system.

## Conclusion

In this paper, developmental advances in the CPS, named I-nteract, have been presented. I-nteract provides a framework to develop intuitive and automated interfaces to allow non-technical consumers to design customized products for personal fabrication. 3D model generation functionality has been enabled, in the VHMR system (I-nteract), by integrating it with CSG and AI to enable a novice user to design 3D models from scratch. The system has been integrated with SR-DNN to infer the 3D model from a single-view 2D image of a physical object. To overcome the limitation of SR-DNN to generate 3D models of a single genus (genus zero), a novel generative DNN (SliceGen) has been proposed and integrated with I-nteract for single-view 3D model reconstruction. The manual 3D model generation has also been facilitated in MR by integrating CSG within the system. The automated generation of a 3D model using DL with CSG as an editing tool has the potential to enable a novice user to design customized 3D models from scratch. Taking advantage of the immersive feature of MR, the system also allows the user to adjust the dimensions of a virtual model with respect to the design constraints in the physical workspace. The efficacy of the generative CAD functionality of the system has been demonstrated by generating a 3D model using CSG, then by generating 3D models of chairs and tables using SR-DNN and SliceGen from the 2D images captured via HoloLens, and finally by resizing the 3D models using hands in an MR environment with respect to the physical workspace. The novel interface presented in this paper has the potential to facilitate the inclusion of a consumer into the design process towards a prosumer-based and, hence, Industry 4.0 manufacturing customisation approach.

## Supporting information

S1 Video A user generating 3D model of a chair using I-nteract 2.0. (MP4)

S2 Video A user resizing the 3D model of a table to fit in the physical workspace using I-nteract 2.0. (MP4)
